# CellCallEXT: Analysis of Ligand–Receptor and Transcription Factor Activities in Cell–Cell Communication of Tumor Immune Microenvironment

**DOI:** 10.3390/cancers14194957

**Published:** 2022-10-10

**Authors:** Shouguo Gao, Xingmin Feng, Zhijie Wu, Sachiko Kajigaya, Neal S. Young

**Affiliations:** Hematopoiesis and Bone Marrow Failure Laboratory, Hematology Branch, National Heart, Lung, and Blood Institute, National Institutes of Health, Bethesda, MD 20892, USA

**Keywords:** single-cell RNA-seq, cell–cell interaction, ligand–receptor–transcription factor axis

## Abstract

**Simple Summary:**

CellCall is an R package tool that is used to analyze cell–cell communication based on transcription factor (TF) activities calculated by cell-type specificity of target genes and thus cannot directly handle two-condition comparisons. We developed CellCallEXT to complement CellCall. CellCallEXT can directly identify ligand–receptor (L–R) interactions that alter the expression profiles of downstream genes between two conditions, such as tumor and healthy tissue. Scoring in CellCallEXT quantitatively integrates expression of ligands, receptors, TFs, and target genes (TGs). The pathway enrichment analysis and visualization modules allow biologists to investigate how disease alters cell–cell communication. Furthermore, Reactome pathways were added into CellCallEXT to expand the L–R–TF database.

**Abstract:**

(1) Background: Single-cell RNA sequencing (scRNA-seq) data are useful for decoding cell–cell communication. CellCall is a tool that is used to infer inter- and intracellular communication pathways by integrating paired ligand–receptor (L–R) and transcription factor (TF) activities from steady-state data and thus cannot directly handle two-condition comparisons. For tumor and healthy status, it can only individually analyze cells from tumor or healthy tissue and examine L–R pairs only identified in either tumor or healthy controls, but not both together. Furthermore, CellCall is highly affected by gene expression specificity in tissues. (2) Methods: CellCallEXT is an extension of CellCall that deconvolutes intercellular communication and related internal regulatory signals based on scRNA-seq. Information on Reactome was retrieved and integrated with prior knowledge of L–R–TF signaling and gene regulation datasets of CellCall. (3) Results: CellCallEXT was successfully applied to examine tumors and immune cell microenvironments and to identify the altered L–R pairs and downstream gene regulatory networks among immune cells. Application of CellCallEXT to scRNA-seq data from patients with deficiency of adenosine deaminase 2 demonstrated its ability to impute dysfunctional intercellular communication and related transcriptional factor activities. (4) Conclusions: CellCallEXT provides a practical tool to examine intercellular communication in disease based on scRNA-seq data.

## 1. Introduction

**Figure 1:**
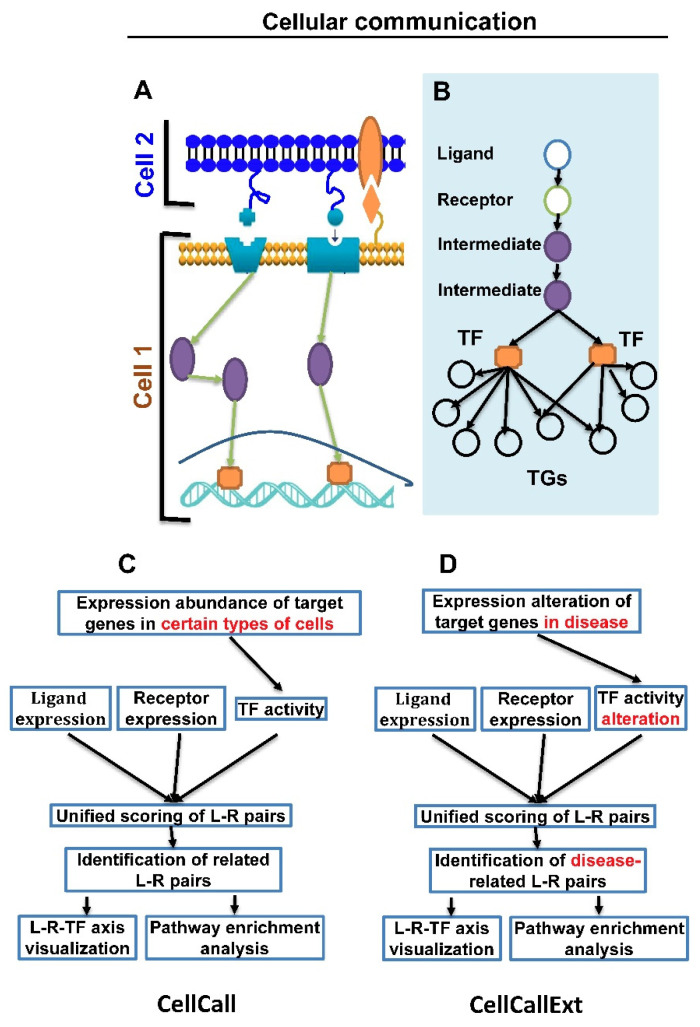
Models and algorithms. (**A**) Cell 1 and Cell 2 communicate through two ligand (L)–receptor (R) pairs from Cell 2 to Cell 1 and one L–R pair from Cell 1 to Cell 2. Binding of ligands with receptors triggers gene expression changes through intermediate genes and transcriptional factors (TFs). (**B**) Biological diagram of signal transduction from a ligand to target genes (TGs). (**C**,**D**) Statistical concepts of CellCall and CellCallEXT algorithms. Three contributions are considered: ligand expression in sender cells, receptor expression in receiver cells, and TF activities in receiver cells. Main difference from CellCall is integration of TF activity alteration between two conditions, instead of TF activity (highlighted in red). Algorithm is implemented in Equations (4)–(6) in Methods.

No metazoan cells survive without communication with other cells [1,2]. Multicellular life relies on cell–cell interactions across diverse cell types. Modeling of cell–cell interactions can also be applied to understand disease mechanisms [1,2,3,4,5,6,7,8,9,10,11,12]. Ligand signaling from sender cells into receiver cells and changes of receiver cells’ expression profiles are often mediated by a series of interactions between ligands (L) and receptors (R), receptors and transcriptional factors (TFs), and their target genes (TGs) (Figure 1A). Although physical cell–cell interaction mapping remains experimentally challenging, cell–cell communication can be inferred from single-cell transcriptomics data [2]. Many algorithms such as CellPhoneDB, NicheNet, SingleCellSignalR, and CellCall [1,3,4,11] have been applied to infer links between ligands and receptors, as well as TFs and TGs. These programs can be divided into two categories: the first category is based on gene expression abundance in different types of cells, and the second on gene expression alteration in disease.

CellphoneDB [2,13,14] is an online tool working with Python. The algorithm scores ligand–receptor (L–R) pairs by the *p*-values of the mean score to infer potential L–R relationships. CellphoneDB is based on gene expression abundance in different types of cells. The limitation of CellphoneDB is that high expression of ligands and receptors at RNA levels does not necessarily mean that they interact in reality. The actual interaction more likely happens when downstream signaling effects from such L–R interactions are observed. Associated TF activities are direct indicators of effects from L–R pairs, but they are not included in the algorithm of CellphoneDB.

NicheNet complements CellphoneDB. It is based on gene expression alterations in a disease [1]. NicheNet is designed to predict ligand–target gene links between cells by combining expression data with prior knowledge of extracellular signaling and downstream gene regulatory relationships. A merit of NicheNet is the established integrated network, which comprises L–R interactions, intracellular signaling, and gene regulatory interactions. NicheNet aims to find L–R pairs that are likely to lead to gene expression changes in receiver cells in disease rather than prioritizing L–R pairs based on expression abundance. The input of NicheNet is an interesting gene set, usually differentially expressed genes in receiver cells identified in a disease state, which is integrated into the prior network knowledge for L–R prioritization. Gene expression levels are used to infer a disease-affected gene list and determine whether the expression levels of ligands and receptors are high enough to be considered significant. However, expression values are not quantitatively integrated into the algorithm.

CellCall is an algorithm of the first category that quantitatively utilizes gene expression data. It is a new tool to infer intercellular communication by combining ligand–receptor expression and downstream TF activities for a given L–R pair. The algorithm not only accounts for intracellular signaling but also offers a threshold for intercellular TF activities, a more reasonable strategy than simply assessing the expression intensity and/or specificity of the L–R pair. CellCall first calculates fold changes (FCs) of the gene expression of TGs between a given cell type and all other cells, which reflects their expression specificity in the given cell types. The gene set enrichment analysis (GSEA) is used on FC to obtain a normalized enrichment score (NES) that represents TF activity. Similar with CellphoneDB, CellCall belongs to the first category of the algorithm and utilizes cell-type-specific gene expression programs. There is a high risk that ligands will be falsely linked to some cell-intrinsic genes because the cell-type-specific genes are assumed to be induced by cell–cell interactions with other cell types [15].

In addition to imputing inter- and intracellular communication by integrating paired L–R and TF activities, CellCall uses an embedded pathway activity analysis method to identify significantly activated pathways involved in intercellular crosstalk between certain cell types. It has three limitations: (1) CellCall cannot cope with gene expression changes in a disease, as an L–R–TF model with cell-type specificity is only one component of intercellular signal transduction. (2) CellCall only includes KEGG pathways, which are mostly metabolic. (there are only limited numbers of other types of pathways). (3) Since it is designed for steady-state conditions, it considers the more active TFs in only certain cell types.

To address these problems, we have developed a computational method called CellCallEXT by extending interaction databases that made it applicable to gene expression alterations in a disease [16]. This tool appears a good complement to CellCall. CellCallEXT was designed to identify the L–R interactions that altered the expression profiles of downstream pathophysiologic genes rather than the L–R interactions with gene expression in cell populations in a healthy status under a steady condition. The main modules included (1) identification of altered L–R pairs, (2) heatmap plots of L–R alterations, and (3) pathway analysis and visualization related to TGs. In the following sections, the method and applications in the studies of a genetic syndrome, DADA2 and cancer were described. Finally, the main features and limitations of this tool were summarized.

## 2. Materials and Methods

An intercellular communication model and a pipeline algorithm of CellCallEXT are shown in Figure 1. Cell–cell communication is initiated with binding ligands to receptors. Intercellular L–R interactions provide bridges for signal transfer between sender cells and receiver cells. Binding of a ligand with a receptor changes the conformation of a receptor and subsequently leads to perturbed expression levels of downstream TFs and their TGs (Figure 1A,B).

In the CellCall package, L–R–TF axes and TF–TG interaction data were collected. L–R–TF axis datasets were extracted from the KEGG pathway analysis using the following steps: (1) Human L–R interactions were obtained from the NATMI, Cellinker, CellTalkDB, CellChat, and STRING databases; ligand–receptor complexes were included among the retrieved L–R interactions. (2) TFs downstream of the L–R interactions from the KEGG pathways were extracted. Only the L–R interactions and downstream TFs in the same branch of a given pathway were identified as an L–R–TF axis, and distances between receptors and TFs were calculated. The current version includes 19,144 human L–R–TF axes. Human TF–TG interactions (587248) were collected from TRANSFAC, JASPAR, and 10 other databases. The KEGG database has a very limited number of signal-transduction-related maps important for cell–cell communication. We extended the data sources by adding Reactome into the L–R–TF axis dataset. Reactome pathways were included for enrichment analysis [17].

### 2.1. Inferring Intercellular Communication

Based on the biological model of Figure 1A,B, we built a statistical model for an intercellular communication with three parts: ligand expression, receptor expression, and a regulon activity change (Figure 1C). A regulon was a set of TGs for a TF coexpressed with the TF. Cell–cell communication of an L–R pair was calculated as a unified score from ligand and receptor expression, and intracellular TF activity changes (scores of downstream TFs) in the receiver cells. GSEA was used to score TFs. CellCallEXT was able to quantify intercellular communication changes for certain L–R pairs and to assess L–R internal regulatory signaling changes based on receptor-associated TFs. In addition, CellCallEXT provided two pathway enrichment analyses: the Jaccard coefficient and hypergeometric test.

Ligand expression L is its expression value and is calculated by a geometric mean of the expression values of all subunits if the ligand is a complex containing n subunits:

(1) $$L=\sqrt[{n}]{\overset{n}{\underset{g=1}{\prod }}l_{g}}$$

where $l_{g}$ is an expression value of subunit g in the ligand complex.

Similarly, receptor expression R is its expression value and is calculated by a geometric mean of expression values of all subunits:

(2) $$R=\sqrt[{n}]{\overset{n}{\underset{h=1}{\prod }}r_{h}}$$

where $r_{h}$ is an expression value of subunit h in a receptor complex.

For an L−R interaction k, a TF activity score $TF_{k}$ is assessed according to the expression of the TF regulon. Regulon is a set of TGs of a TF that is coexpressed with the TF across all cells. Its formula is as follows:

(3) $$Regulon=G_{TG}\cap G_{coexp}$$

where $G_{TG}\;$ is a gene set of all $TG_{s}$ for a TF, and $G_{coexp}$ is a gene set of all coexpressed genes of a TF. $\;G_{coexp}$ is selected by the Spearman’s rank correlation coefficient with preset cutoffs ($p<0.05,\;\left|R\right|>0.1$). Cutoffs can be set according to the characteristic of a dataset, such as cell numbers.

A GSEA-NES is used to represent regulon $\;TF_{k,i}$ activity of TF i of L−R interaction k. Its formula is as follows, depending on a pre-chosen interesting expression alteration (up, down, or both):

(4) $$TF_{k,i,up}=\left\{\begin{array}{c} \;0.\;\;\;\;\;\;\;\;\;\;\;\;\;\;\;\;\;\;\;\;\;\;\;\;\;\;\;\;\;\;\;\;\;\;\;\;\;\;\;\;p\geq \alpha \;or\;NES<0\\ GSEA\left(FC,Regulon\right).\;\;\;\;\;\;\;p<\alpha \;and\;NES>0\; \end{array}\right.$$

(5) $$TF_{k,i,down}=\left\{\begin{array}{c} 0.\;\;\;\;\;\;\;\;\;\;\;\;\;\;\;\;\;\;\;\;\;\;\;\;\;\;\;\;\;\;\;\;\;\;\;\;\;\;\;\;\;\;\;\;\;\;p\geq \alpha \;or\;NES>0\\ abs\left(GSEA\left(FC,Regulon\right)\right).p<\alpha \;and\;NES<0\; \end{array}\right.)$$

(6) $$TF_{k,i,both}=\left\{\begin{array}{c} 0.\;\;\;\;\;\;\;\;\;\;\;\;\;\;\;\;\;\;\;\;\;\;\;\;\;\;\;\;\;\;\;\;\;\;\;\;\;\;\;\;\;\;\;\;\;\;\;\;\;\;adjust.p\geq \alpha \\ abs\left(GSEA\left(FC,\;Regulon\right)\right).\;\;\;\;adjust.p<\alpha \end{array}\right.$$

where *FC* is a fold change between a disease and control samples of all $TG_{s}$ in the regulon, and p is a significance level of GSEA, calculated by the clusterProfiler package. If adjust.p is lower than a threshold α (default as 0.05), $TF_{k,i}$ is defined as an absolute value of the NES of GSEA; otherwise, $TF_{k,i}$ is set to 0.

Equations (4)–(6) make CellCallEXT different from CellCall. CellCall firstly calculates the FCs of TGs between one given cell type and all other cells for GSEA calculation. CellCall does not have an option of direction because it only considers expression abundance in certain cell types, instead of expression alterations in a disease.

The activity score $TF_{k}\;$ is defined as a weighted mean of all TFs when there are more than one downstream TF for a L−R interaction k, as follows:

(7) $$TF_{k}=\overset{n}{\underset{i=1}{\sum }}\frac{1/M_{k,i}}{\sum_{i=1}^{n}1/M_{k,i}}\times TF_{k,i}\;$$

where *M* is the shortest step from $TF_{k,i}$ to a receptor k in a pathway, and n is the number of TFs.

Cell–cell communication between different cell types $S_{k}$ is defined as a unified score of an L−R interaction k between cell types i and j, which is calculated by integrating an *L*2 norm of an L−R interaction ${\vec{LR}}_{k}$ and its activity score of the downstream TF $TF_{k}$. Its formula is as follows:

(8) $$S_{k}=\vec{\|LR_{k}\|{}_{2}}\times TF_{k}\;\;\;$$

where $\vec{\|LR_{k}\|{}_{2}}$ is calculated by normalized expression values of a ligand and a receptor for the L−R interaction k:

(9) $$\vec{\|LR_{k}\|{}_{2}}=(softmax\left(L_{i,k}\right),\;softmax\left(R_{j,k}\right)\;$$

where $L_{i,k}$ is a mean expression value of the ligand in a cell type i, and $R_{j,k}$ is a mean expression value of the receptor in cell j. The expression values of the ligand and receptor can be recalculated with Equations (1) and (2) when they contain subunits. A quantile expression value of the ligand–receptor to represent $L_{i,k}$ and $R_{j,k}$ can be chosen to lower the influence of the dropout of the scRNA-seq data [11].

### 2.2. Pathway Enrichment Analysis

CellCallEXT includes pathway enrichment analysis to identify pathways involved in cell–cell communication alterations. In CellCallEXT, the enrichment of pathway i is based on the Jaccard overlap combined coefficient, defined as

(10) $$PAS_{i}=\frac{C_{LR}\cap P_{LR}}{C_{LR}\cup P_{LR}}\;$$

where $C_{LR}$ is the L−R interaction between certain cell types inferred by communication analysis. $P_{LR}$ is all the L−R interactions in a pathway. Then, a z-score-normalized score is calculated as follows:

(11) $$nPAS_{i}=\frac{PAS_{i}-\bar{PAS_{i}}}{\sigma }$$

Pathway enrichment is also assessed by hypergeometric testing to estimate significance. Its formula is as follows:

(12) $$P=1-\overset{q-1}{\underset{k=0}{\sum }}\frac{\left(\begin{array}{c} t\\ k \end{array}\right)\left(\begin{array}{c} m-t\\ n-k \end{array}\right)}{\left(\begin{array}{c} m\\ n \end{array}\right)}$$

where t is the number of L−R interactions inferred by communication analysis between two cell types, and  n is the number of L−R interactions in a pathway. m is the number of all L−R interactions. q is an intersect of t and n.

### 2.3. Data Collection and Processing of scRNA-seq Datasets

Eight processed tumor immune microenvironment (TIME) scRNA-seq datasets were collected from the TISCH database: GSE114727, GSE139555 (kidney renal clear cell carcinoma (KIRC), colorectal cancer (CRC), and non-small cell lung cancer (NSCLC)), GSE146771, GSE116256, GSE140228, and GSE117570 [13]. A standardized analysis workflow based on MAESTRO v1.1.0 was applied for a quality control, batch effect removal, cell clustering, and cell-type annotation based on the expression matrix, with expression in each cell scaled to 10,000. Though CellCallEXT was designed for the TIME, it could be used to analyze the microenvironments of other diseases. To demonstrate this, scRNA-seq data of ~180,000 human CD3^+^ T cells and CD14^+^ monocytes from 10 deficiency of adenosine deaminase 2 (DADA2) patients and five healthy donors were collected from our previous studies (GSE168163 and GSE142444), and their expression levels were normalized by log2[CPM/10 + 1] (counts per million, CPM), with scale factors of 10,000 [8,12].

## 3. Results

CellCallEXT complemented CellCall in using gene expression to examine cell communication. The algorithm estimated the activity changes of TFs. As shown in Figure 1C, a unified score was calculated from three components: ligand expression, receptor expression, and TF activity alteration. Most functions to visualize results in CellCall were applicable to CellCallEXT, with slight modifications.

### 3.1. Comparison of CellCallEXT with Other Tools

The general features of CellCall compared with nine other tools in three aspects (data, approach, and visualization) have been described [11]. CellCall collected 19,144 L–R–TF axes from KEGG pathway analysis. CellCallEXT included all L–R–TF axes in CellCall and added 24,649 axes retrieved from Reactome. Over 2000 further pathways were added into the library. There were many redundant pathways in Reactome, and we will merge or remove the redundancies in the future. The general features of CellCallEXT and other several tools are shown in Table 1.

### 3.2. Inferring Cell–Cell Communication in TIME

**Figure 2:**
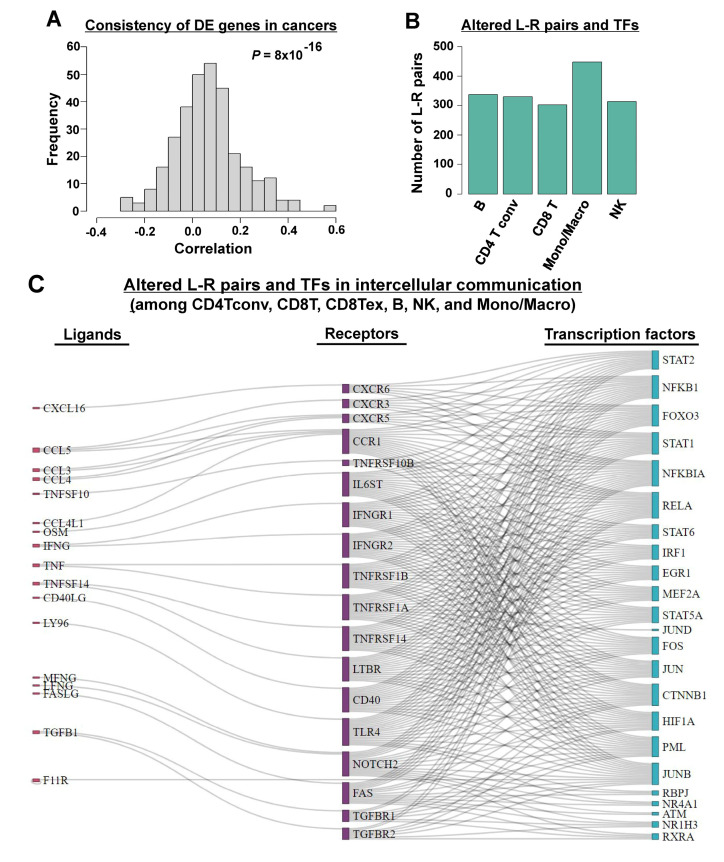
Commonly altered ligand (L)—receptor (R)–transcription factor (TF) communication in cancers of tumor immune microenvironments (TIMEs). (**A**) Consistency of differentially expressed genes in different cancers of TIME. (**B**) The number of identified L—R pairs by senders or receivers in eight TIME datasets, grouped by cell types. (**C**) Sankey plot of common altered L—R pairs and downstream TFs for intercellular communication among conventional CD4^+^ T cells (CD4Tconv), CD8^+^ T cells (CD8T), exhausted CD8^+^ T cells (CD8Tex), B cells (**B**), natural killer cells (NK), and monocytes and macrophages (Mono/Macro) cells in seven TIME datasets.

Intercellular crosstalk between immune cells in the tumor niche links inflammation, immunity, and tumorigenesis. Here, we applied CellCallEXT to eight TIME scRNA-seq datasets, which comprised five types of cancers, including one acute myeloid leukemia dataset, two NSCLC datasets, one KRIC dataset, two CRC datasets, and one breast invasive carcinoma dataset. All datasets included both tumor and normal samples. First, intercellular communication among six immune cell types, namely B cells (B), conventional CD4^+^ T cells (CD4Tconv), CD8^+^ T cells (CD8T), exhausted CD8^+^ T cells (CD8Tex), monocytes and macrophages (Mono/Macro), and natural killer (NK) cells, was analyzed by CellCallEXT. We first checked the commonality of expression changes in different types of cancers by calculating FCs between tumor and healthy donor samples in different cell populations and then performed pairwise correlation of FCs across datasets in the same cell population. We found that there was higher correlation of FCs across different datasets, indicating that different cancers shared similar gene expression profiles and some common mechanisms (Figure 2A).

The identified L–R pairs and related pathways for all cancer types are given in the supplementary files, and we reported the common L–R–TFs across all types of cancer here.

Twenty-five common tumor-specific intercellular communications were identified in more than four datasets (Supplementary Figure S1), mainly involved in intercellular communication from other cells to Mono/Macro, including CCL3/4/5-CCR1/5 and TNF–TNFRSF1B signaling [16]. C–C motif chemokines (CCL3/4/5) secreted in the TIME play important roles in Mono/Macro differentiation, activation, polarization, and recruitment by binding specific C–C motif chemokine receptors (CCR1/5). TNF–TNFRSF1B signaling plays a central role in the negative regulation of M2 tumor-associated macrophages. As shown in Supplementary Figures S2–S18, compared with other cell types, Mono/Macro cells received significantly more signals from other immune cells and sent significantly more signals to other immune cells across all datasets (Figure 2B, Supplementary Figure S2), indicating dominant roles for Mono/Macro in the intercellular crosstalk of immune cells in the TIME. The same observation was also made using CellCall [11]. Tumor-associated macrophages create an immunosuppressive tumor microenvironment (TME) by producing cytokines, chemokines, and growth factors, and triggering inhibitory immune checkpoint protein release in T cells [18]. We also investigated TFs-activated downstream of the communication. Most of the activated TFs are involved in cancer progression through the TIME (Figure 2C, Supplementary Figure S1), such as the NFκB family (*NFKB1*, *NFKBIA,* and *RELA*) and the STAT family (*STAT1*, *STAT2,* and *STAT6*), which are critical in M1 and M2 macrophage polarization [19]. To confirm the roles of these TFs in cancers and test the capacity of CellCallEXT, pathway enrichment analysis was conducted (Supplementary Figure S1). Most identified pathways were related to cancers or associated with tumor growth. For example, IFN-γ associates with tumor growth [20]. IFN-γ is conventionally recognized as a central inflammatory cytokine in the TME [21]. CellCallEXT was able to effectively impute crucial intercellular communication of TIME and discover underlying intracellular processes affected by intercellular crosstalk.

TNF signaling pathways were identified in almost all cancers by CellCallEXT (Supplementary Figures S1–S18). A growing body of epidemiological and clinical data supports the concept that chronic inflammation promotes tumor development and progression. As a major proinflammatory cytokine, *TNF* acts as an endogenous tumor promoter, bridging inflammation and carcinogenesis. *TNF* is involved in all aspects of carcinogenesis including cellular transformation, survival, proliferation, invasion, angiogenesis, and metastasis. *TNF* is secreted by inflammatory cells and functions by activating signaling pathways, such as NFκB and c-Jun N-terminal kinase (*JNK*). *NFκB* is a major anti-apoptotic cell survival signal, and sustained JNK activation contributes to cell death. The crosstalk between the *NFκB* and JNK determines cellular outcomes in response to *TNF*. *TNF* is an endogenous tumor promoter because of its stimulatory effects on cancer cell growth, proliferation, invasion, metastasis, and tumor angiogenesis. Conversely, *TNF* induces cancer cell death and has been proposed as a potential cancer therapeutic agent [22,23]. Other common pathways identified by CellCallEXT were cytokine signaling in the immune system, chemokine signaling pathway, diseases pathway, immune system pathway, signaling by interleukins pathway, pathways in cancers, etc. (Supplementary Figures S1–S18), all of which are important in cancer microenvironments [24].

### 3.3. Inferring Cell–Cell Communication in DADA2

We chose DADA2 as a disease model to test CellCallEXT. DADA2 is a monogenic vasculitis syndrome caused by autosomal-recessive loss-of-function mutations in the *ADA2* gene. ADA2 is primarily secreted with stimulated monocytes and macrophages. DADA2 causes abnormal, unprovoked inflammation that can damage diverse tissues and organs, particularly blood vessels. Both CD8^+^ and CD4^+^ T cells are activated in DADA2 patients [25].

Inflammation and immune responses require communication among various types of immune cells. Having identified disrupted gene programs and activation of T cells and monocytes in our previous work [8,12], we integrated data from 10 patients and five healthy donors with paired T-cell and monocyte mRNA profiling in order to examine cell–cell interactions potentially involved in DADA2 pathogenesis [26].

**Figure 3:**
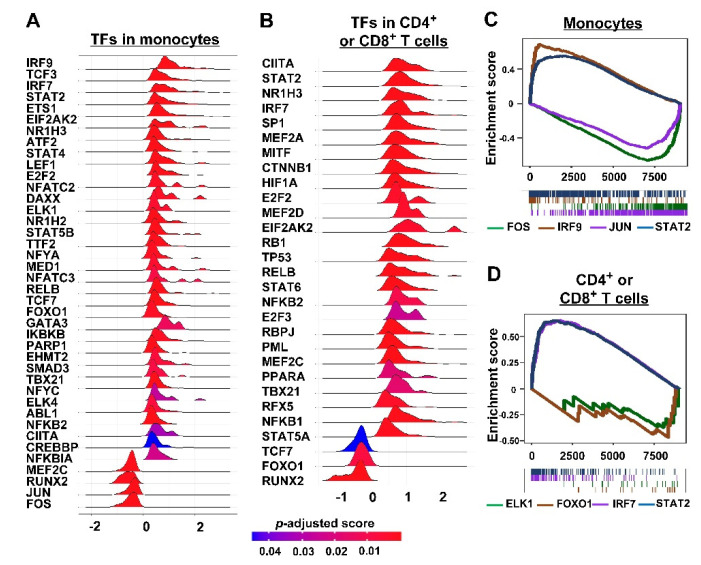
Selected transcription factors (TFs) to score ligand—receptor pairs. Ridge plots of density distributions of fold changes of top TFs for TFs in monocytes (**A**) and CD4^+^ or CD8^+^ T cells (**B**). Enrichment analysis results of target genes of samples with significant TFs in monocytes (**C**) and CD4^+^ or CD8^+^ T cells (**D**).

Among dysregulated TFs, 36 TFs in T cells and 40 TFs in monocytes were identified in their TGs (Figure 3A,B), in which 19 shared TFs were found in the two cell populations. Some TFs are important for inflammation. Several members of the STAT protein family, in particular STAT1, STAT2, STAT3, STAT4, and STAT6, act as TFs in modulating pro- and anti-inflammatory responses. There is abundant evidence for the involvement of the different STAT proteins in inflammation, autoimmune, and allergic diseases [27]. The transcription factor NFKB2 plays an important role in regulating the expression of cytokines in human monocytes. FCs of TGs of several sample TFs are shown in Figure 3C,D: TFs with larger *p* values were excluded for calculation to filter noises, as shown in Equation (8).

**Figure 4:**
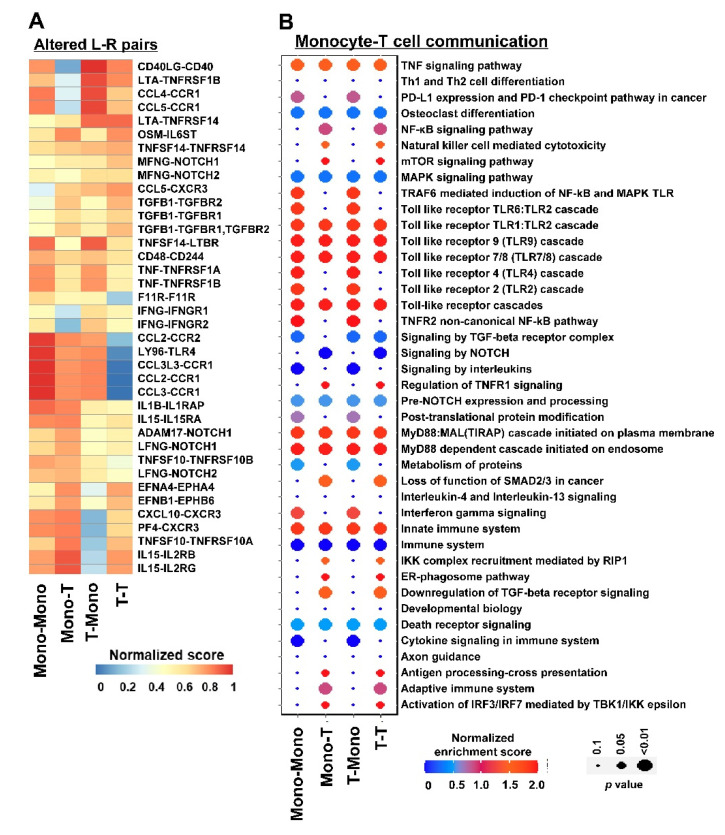
Ligand (L)—receptor (R) pairs and pathways affected by DADA2. (**A**) Significantly altered L–R pairs in intercellular communication between monocytes and T cells. (**B**) Pathway activity analysis of intercellular communication between monocytes and T cells in DADA2 patients. Mono-Mono, monocytes–monocytes; Mono—T, monocytes—T cells; T—Mono, T cells—monocytes; T—T, T cells—T cells.

By CellCallEXT, 39 L–R interactions were identified between T cells and monocytes based on predefined molecular interactions and gene expression changes between DADA2 patients and healthy donors (Figure 4A). Some interactions were only identified in certain cell types. For example, IFNG–IFNGR1 and IFNG–IFNGR2 interactions were only altered between monocytes and monocytes, not between T cells and T cells (Figure 4A). IL15–IL2RGB and IL15–IL2RG only showed alterations from monocytes to T cells, but not from T cells to monocytes. CD40LG–CD40 showed alterations from T cells to monocytes, but not from monocytes to T cells (Figure 4A). CCL2–CCR1 and CCL3–CCR1 did not change in T cells.

**Figure 5:**
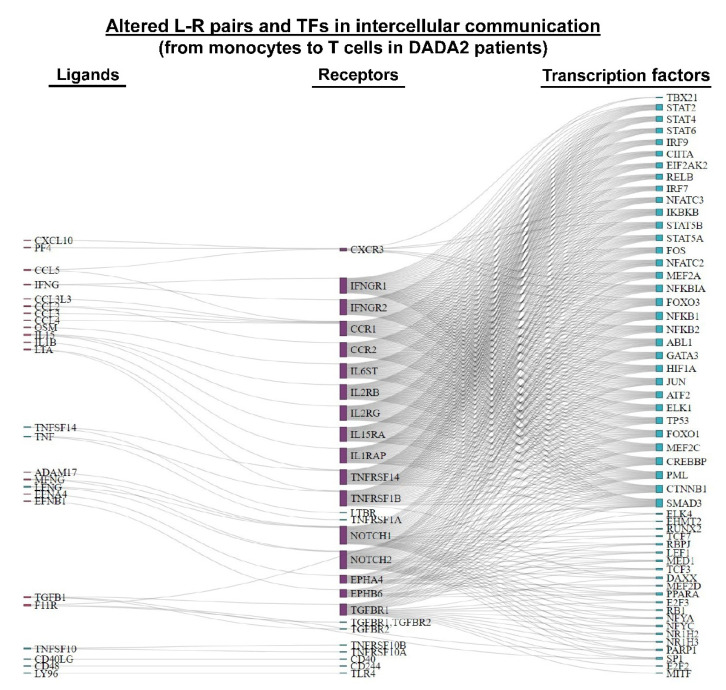
Altered ligand (L) —receptor (R) —transcription factor (TF) communication in DADA2 patients. Sankey plot of significantly altered L—R pairs and downstream TFs in intercellular communication between monocytes and T cells in DADA2 patents.

The results of pathway activity analysis show that intercellular signaling from monocytes and T cells was mainly enriched in inflammation pathways (Figure 4B). These pathways have been reported to be critical in DADA2 pathophysiology. A total of 47 intercellular communication pathways were identified. As shown in a Sankey plot (Figure 5), TFs downstream of the intercellular communication were related to inflammation and development. Some genes were cancer-related, indicating potential relationships of DADA2 and malignancy. Mitogen-activated protein kinases (MAPKs) signaling and NF-kappa B signaling pathways have been reported to play important roles in the DADA2 disease. MAPKs are critical in regulating the production of proinflammatory cytokines and downstream signaling events leading to inflammation. Potential roles and mechanisms of these communications in DADA2 deserve further elucidation [28].

### 3.4. Comparison of DADA2 Results between CellCallEXT and NicheNet

Because CellCall is based on gene expression levels while CellCallEXT is based on TGs’ gene expression alterations under a disease, and CellPhoneDB, SingleCellSignalR, and other tools do not include the information of TGs (rows 3 and 6 of Table 1 in [1]), it is not meaningful to compare these toolkits. Instead, we only compared the results between CellCallEXT and NicheNet because both are based on gene expression changes in a disease (Supplementary Figures S19–S21). Five overlapping L–R pairs were found, much higher than expected by chance (*p* value of 0.001 (hypergeometric test)). Both methods integrate gene expression alterations and ligand and receptor expression. After careful examination, shared L–R pairs had high weights in NicheNet, suggesting consistency of results when using both methods together, but each could also provide distinct information, analogous to the Fisher test (with a set of interesting genes) and GSEA (with quantitative information of gene expression) in pathway analysis. Another advantage of CellCallEXT is its identification of enriched pathways, not available in NicheNet. Biologists can interpret the changes in cell–cell communication in a disease.

## 4. Discussion

Cells communicate by sending and receiving signals. In order to trigger responses, these signals must be transmitted across the cell membranes. Investigation of intercellular communication alterations should facilitate the understanding of pathogenic mechanisms in a disease. Here, we extended CellCall by adding Reactome datasets and modifying the algorithm, and thus created a toolkit to examine perturbed intercellular communication. Successful application of our algorithm to monocytes and T cells of patients with DADA2 and of the TIME from cancer patients demonstrate CellCallEXT can effectively infer altered intercellular communication and internal signaling under physiological and pathological conditions. Conceptionally, CellCallEXT is similar to NicheNet, but the former utilizes the quantitative expression information of ligands, receptors, TFs, and TGs. As does NicheNet, CellCallEXT uses gene expression for ligands and receptors, rather than expression alterations in a disease, on the assumption that only results from highly expressed L–R interactions are reliable. CellCallEXT should not be considered an improvement on CellCall, as they belong to different categories of tools for cell–cell communication analysis; they complement each other in addressing different biological questions. Although many computational instruments have been developed and extensively applied in studies of receptors and ligands, a common problem is their reliance on the database of known L–R pair interactions, which is still relatively under developed, especially for condition-specific L–R pairs [29]. A comprehensive and reliable resource of L–R pairs is needed to cover more information of receptors, ligands, TFs, and their interactions for the development and assessment of new and existing tools [30].

## 5. Conclusions

CellCallEXT should enable examination of intercellular communication in diseases from scRNA-seq data. CellCallEXT is theoretically similar to NicheNet, but has specific advantages: (1) identification of enriched pathways in order to interpret disease-specific changes in cell–cell communication; and (2) the scoring approach quantitatively integrates expression information from ligands, receptors, TFs, and TGs.

As a complement to CellCall, CellCallEXT entails gene expression changes in disease and extends the L–R–TF datasets and pathway information with Reactome repertories, which include more signaling pathways than the KEGG database.

For the evaluation of software applicable to cell–cell interactions, especially of the second type (identifying L–R alterations in disease), there is no gold standard to evaluate their performance. To date, only scattered L–R pairs have been experimentally identified. Comprehensive collection and annotation of L–R–TF datasets are critical and will be useful. Pathway analysis can only provide indirect validation. Intracellular signaling involves multiple protein modifications and interactions, rather than changes in gene expression. Genome-level protein expression more directly addresses L–R interactions for cell–cell communication [31]. Inferred alterations of L–R pairs from transcriptomics may not coincide with proteomic data. CITE-seq, which couples scRNA-seq with protein measurements, may provide important information for cell–cell interaction research [32].

## Figures and Tables

**Table 1:** Feature comparisons among five tools.

Title 1	CellCallEXT	CellCall	NicheNet	CellPhoneDB	SingleCellSignalR
Ligand	Expression value	Expression value	If expressed (Boolean)	Expression value	Expression value
Receptor	Expression value	Expression value	If expressed (Boolean)	Expression value	Expression value
Target genes	Expression alteration by disease	Expression abundance	Expression alteration by disease	Not considered	Not considered
Data size	43,793 L–R–TF	19,144 L–R–TF	12,019	1396	3251

## Data Availability

The tool and sample script are available at https://github.com/shouguog/cellcallEXT.

## Supplementary Materials

The following supporting information can be downloaded at: https://www.mdpi.com/article/10.3390/cancers14194957/s1. Figure S1. Common ligand (L)–receptor (R) pairs in intercellular communication among tumors and immune cells, and common pathways enriched in identified L–R pairs. Figure S2. Ligand (L)–receptor (R) pairs by sender–receiver, a sender only, or a receiver only. Figures S3–S18. Ligand (L)–receptor (R) pairs by sender–receiver, and pathway activities in different cancers. Figure S19. Ligand (L)–receptor (R) pairs in intercellular communication in DADA2 patients identified by NicheNet and CellCallEXT. Figure S20. Sankey plot of altered ligand (L)–receptor (R) pairs and downstream transcription factors (TFs) in intercellular communication from T cells to monocytes of DADA2 patents. Figure S21. Sankey plot of altered ligand (L)–receptor (R) pairs and downstream transcription factors (TFs) in intercellular communication from monocytes to T cells of DADA2 patents.
