# Supplementary material for: CellCallEXT: Analysis of Ligand–Receptor and Transcription Factor Activities in Cell–Cell Communication of Tumor Immune Microenvironment
Source: Cancers (Basel). 2022 Oct 10;14(19):4957. doi: 10.3390/cancers14194957 (PMC9563271; doi:10.3390/cancers14194957)
Supplement: Supplementary file 1 [file cancers-14-04957-s001.zip › cancers-1914764-supplementary.pdf]

CellCallEXT: Analysis of Ligand–Receptor and Transcription Factor Activities in Cell–Cell Communication of Tumor Immune Microenvironment

Shouguo Gao *, Xingmin Feng , ZhijieWu , Sachiko Kajigaya and Neal S. Young

Hematopoiesis and Bone Marrow Failure Laboratory, Hematology Branch, National Heart, Lung, and Blood Institute, National Institutes of Health, Bethesda, MD 20892, USA

* Correspondence: shouguo.gao@nih.gov

Supplementary Figures

Contents

Supplementary Fig. S1. Common ligand (L)-receptor (R) pairs in intercellular communications among tumors and immune cells, and common pathways enriched in identified L-R pairs.

Supplementary Fig. S2. Ligand (L)-receptor (R) pairs by sender-receiver, sender only or receiver only.

Supplementary Fig. S3. Altered ligand (L)-receptor (R) pairs among cell populations in tumor-specific intercellular communications in BRCA_GSE114727.

Supplementary Fig. S4. Pathway activities in intercellular communications among cell populations of BRCA_GSE114727.

Supplementary Fig. S5. Altered ligand (L)-receptor (R) pairs among cell populations in tumor-specific intercellular communications in CRC_GSE139555:

Supplementary Fig. S6. Pathway activities in intercellular communications among cell populations of CRC_GSE139555.

Supplementary Fig. S7. Altered ligand (L)-receptor (R) pairs among cell populations in tumor-specific intercellular communications in CRC_GSE146771_Smartseq2.

Supplementary Fig. S8. Pathway activities in intercellular communications among cell populations of CRC_GSE146771_Smartseq2.

Supplementary Fig. S9. Altered ligand (L)-receptor (R) pairs among cell populations in tumor-specific intercellular communications in KIRC_GSE139555.

Supplementary Fig. S10. Pathway activities in intercellular communications among cell populations of KIRC_GSE139555.

Supplementary Fig. S11. Altered ligand (L)-receptor (R) pairs among cell populations in tumor-specific intercellular communications in AML_GSE116256.

Supplementary Fig. S12. Pathway activities in intercellular communications among cell populations of AML_GSE116256.

Supplementary Fig. S13. Altered ligand (L)-receptor (R) pairs among cell populations in tumor-specific intercellular communications in LIHC_GSE140228_Smartseq2.

Supplementary Fig. S14. Pathway activities in intercellular communications among cell populations of LIHC_GSE140228_Smartseq2.

Supplementary Fig. S15. Altered ligand (L)-receptor (R) pairs among cell populations in tumor-specific intercellular communications in NSCLC_GSE117570.

Supplementary Fig. S16. Pathway activities in intercellular communications among cell populations of NSCLC_GSE117570.

Supplementary Fig. S17. Altered ligand (L)-receptor (R) pairs among cell populations in tumor-specific intercellular communications in NSCLC_GSE139555.

Supplementary Fig. S18. Pathway activities in intercellular communications among cell populations of NSCLC_GSE139555.

Supplementary Fig. S19. Ligand (L)-receptor (R) pairs in intercellular communications in DADA2 patients identified by NicheNet and CellCallEXT.

Supplementary Fig. S20. Sankey plot of altered ligand (L)-receptor (R) pairs and downstream transcription factors (TFs) in intercellular communications from T cells to monocytes of DADA2 patents.

Supplementary Fig. S21. Sankey plot of altered ligand (L)-receptor (R) pairs and downstream transcription factors (TFs) in intercellular communications from monocytes to T cells of DADA2 patents.

A Intercellular communications

Common L-R pairs
IFNG-IFNGR1
IFNG-IFNGR2
CCL4L1-CCR1
MFNG-NOTCH2
OSM-IL6ST
F11R-F11R
CXCL16-CXCR6
CCL3-CCR1
CCL4-CCR1
CCL5-CCR1
CCL5-CXCR3
TGFB1-TGFBR1
TGFB1-TGFBR2
CD40LG-CD40
LY96-TLR4
CCL3-CXCR5
CCL4-CXCR5
CCL5-CXCR5
TNF-TNFRSF1A
TNF-TNFRSF1B
TNFSF14-LTBR
TNFSF14-TNFRSF14
LFNG-NOTCH2
FASLG-FAS
TNFSF10-TNFRSF10B

B Enriched in identical L-R pairs

Common pathways
Chemokine signaling pathway
Cytokine signaling in the immune system
Diseases
Immune system
Signaling by interleukins
Pathways in cancers
Herpes simplex virus 1 infection
HIF-1 signaling pathway
Interferon gamma signaling
Interferon signaling
Protein metabolism
Post-translational protein modification
Signaling by NOTCH
Signaling by TGF-beta family members
Signaling by TGF-beta receptor complex
Jak-STAT signaling pathway
Necroptosis
Osteoclast differentiation
PD-L1 expression and the PD-1 checkpoint pathway in cancer
Toxoplasmosis
Adipocytokine signaling pathway
Hepatitis C
Death receptor signalling
Innate immune system
Interleukin-4 and interleukin-13 signaling
MyD88-dependent cascade initiated on endosome
MyD88:MAL(TIRAP) cascade initiated on plasma membrane
Pre-NOTCH expression and processing
Toll-like receptor cascades
Toll-like receptor 2 (TLR2) cascade
Toll-like receptor 4 (TLR4) cascade
Toll-like receptor 7/8 (TLR7/8) cascade
Toll-like receptor 9 (TLR9) cascade
Toll-like receptor TLR1:TLR2 cascade
Toll-like receptor TLR6:TLR2 cascade
TRAF6 mediated induction of NFkB and MAP kinases upon TLR7/8 or 9 activation
Human cytomegalovirus infection
Human T-cell leukemia virus 1 infection
Insulin resistance
MAPK signaling pathway
Pathogenic Escherichia coli infection
Th1 and Th2 cell differentiation
Th17 cell differentiation
TNF signaling pathway

Fig. S1. (**A**) Common ligand (L)-receptor (R) pairs in the intercellular communications among tumors and immune cells. (**B**) Common pathways enriched in identified L-R pairs.

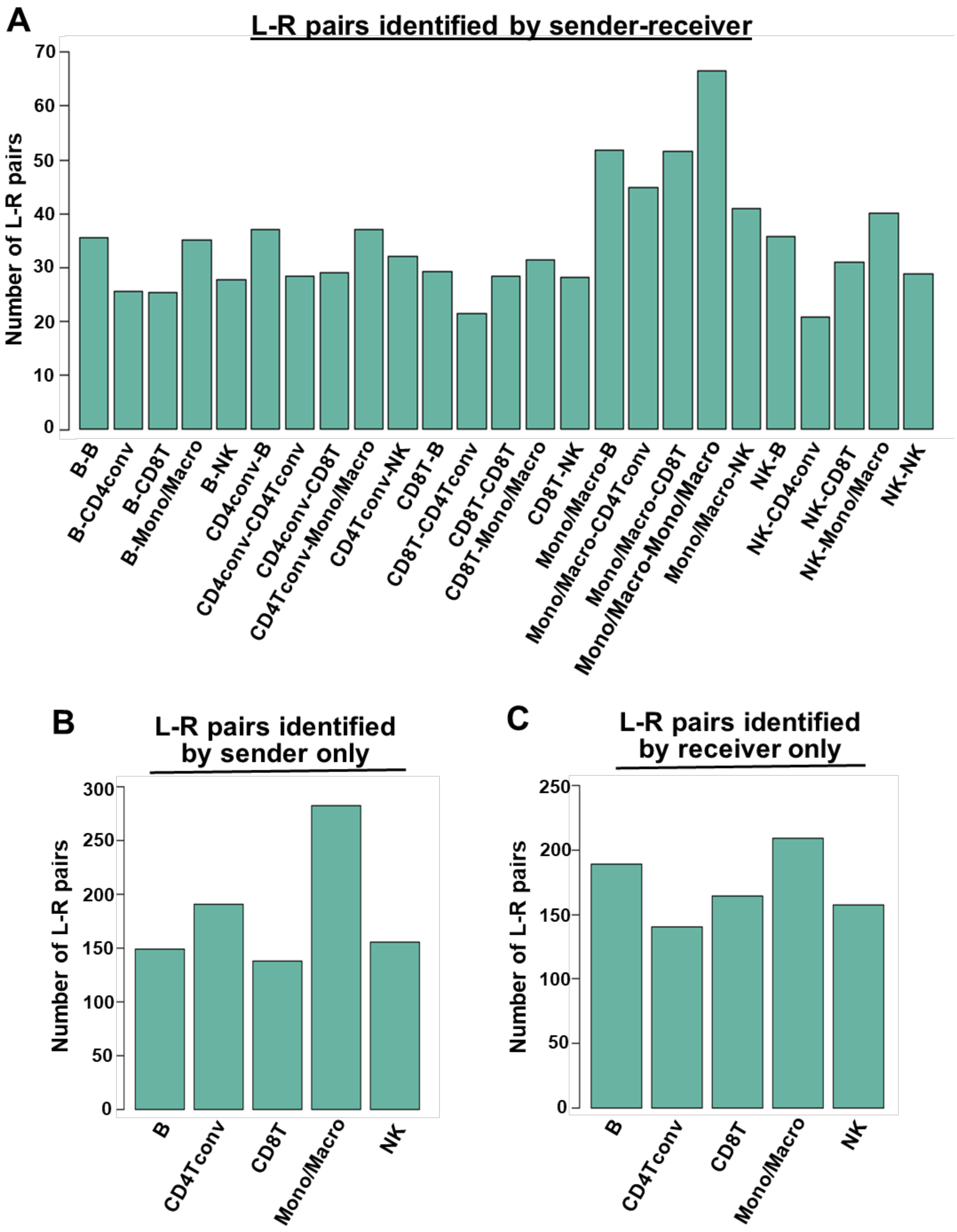

Fig. S2. Ligand (L)-receptor (R) pairs by sender-receiver (**A**), sender only (**B**) or receiver only (**C**): conventional $CD4^+$ T cells (CD4Tconv), $CD8^+$ T cells (CD8T), monocytes/macrophages (Mono/Macro), B cells (B) and natural killer cells (NK).

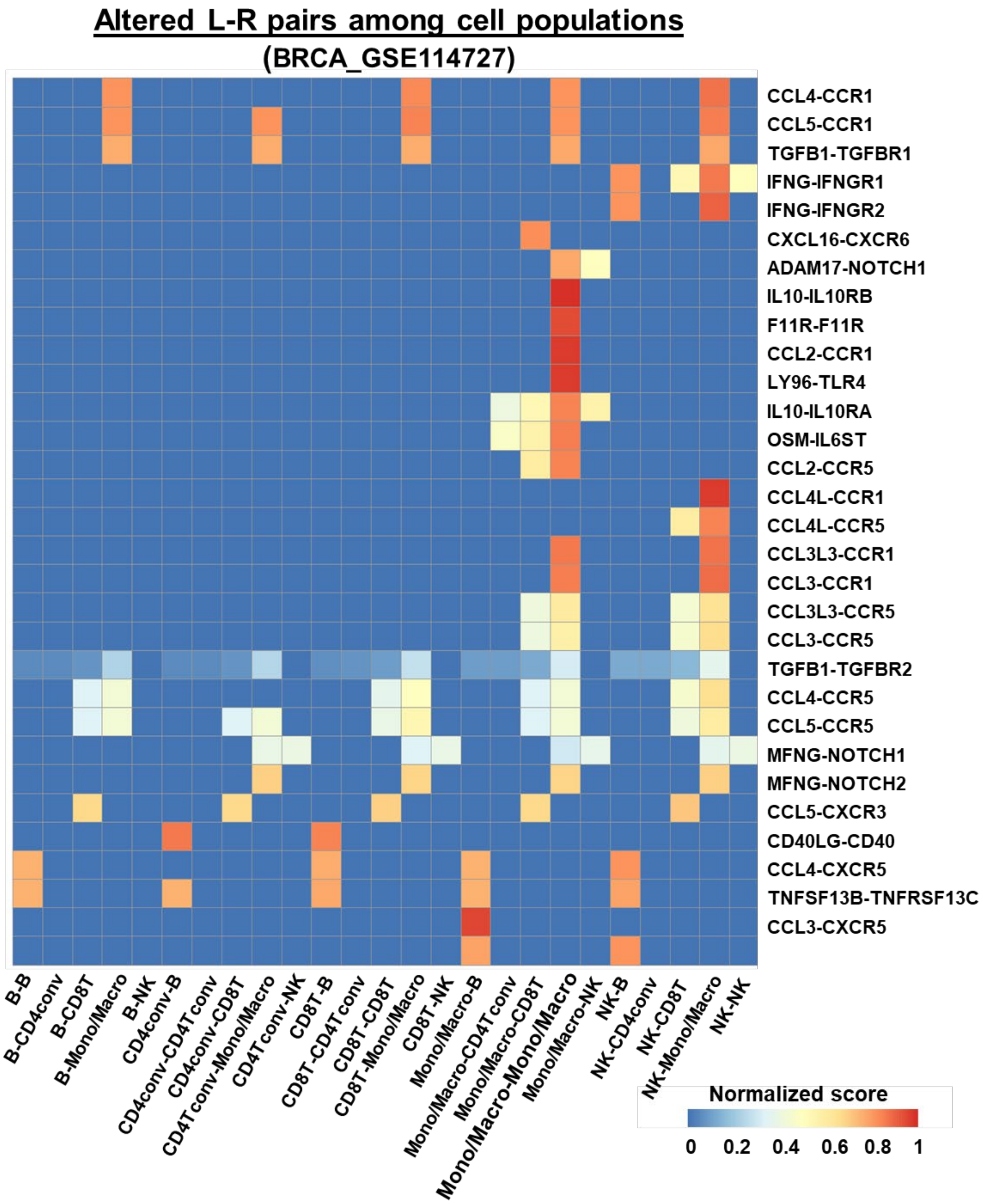

Fig. S3. Altered ligand (L)-receptor (R) pairs among cell populations in tumor-specific intercellular communications in BRCA_GSE114727: conventional $CD4^+$ T cells (CD4Tconv), $CD8^+$ T cells (CD8T), monocytes/macrophages (Mono/Macro), B cells (B) and natural killer cells (NK). Only transcription factors with GSEA *P*-adjusted values < 0.1 were kept for analysis. A cutoff to define correlated target genes for regulon was denoted as a correlation coefficient = 0.1.

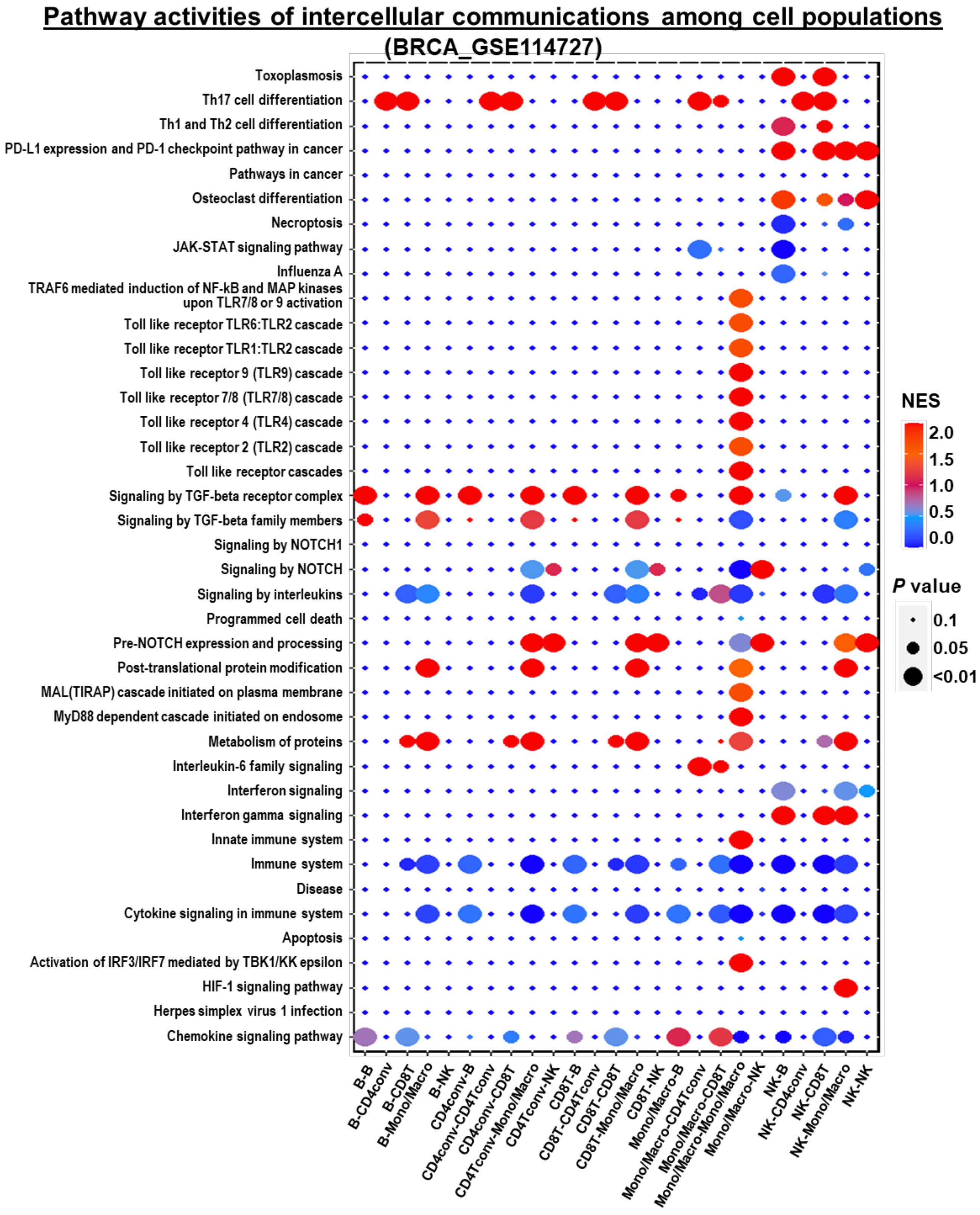

Fig. S4. Pathway activities of intercellular communications among cell populations of BRCA_GSE114727: conventional CD4^{+} T cells (CD4Tconv), CD8^{+} T cells (CD8T), monocytes/macrophages (Mono/Macro), B cells (B) and natural killer cells (NK). Only pathways with $P < 0.1$ in at least one cell populations were retained for analysis. NES, normalized enrichment score.

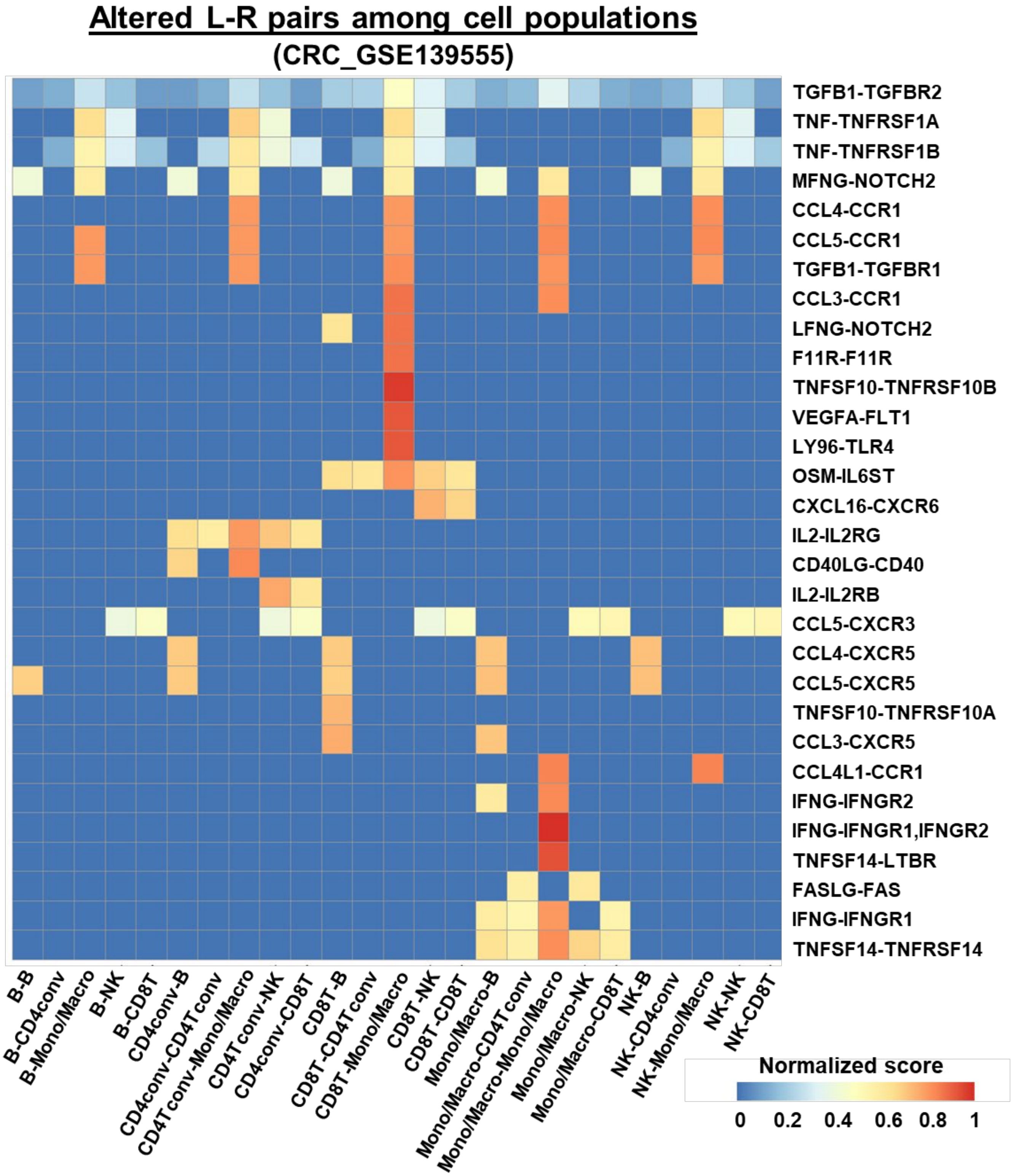

Fig. S5. Altered ligand (L)-receptor (R) pairs among cell populations in tumor-specific intercellular communications in CRC_GSE139555: conventional CD4^{+} T cells (CD4Tconv), CD8^{+} T cells (CD8T), monocytes/macrophages (Mono/Macro), B cells (B) and natural killer cells (NK). Only transcription factors with GSEA *P*-adjusted values < 0.2 were kept for analysis. A cutoff to define correlated target genes for regulon was denoted as a correlation coefficient = 0.02.

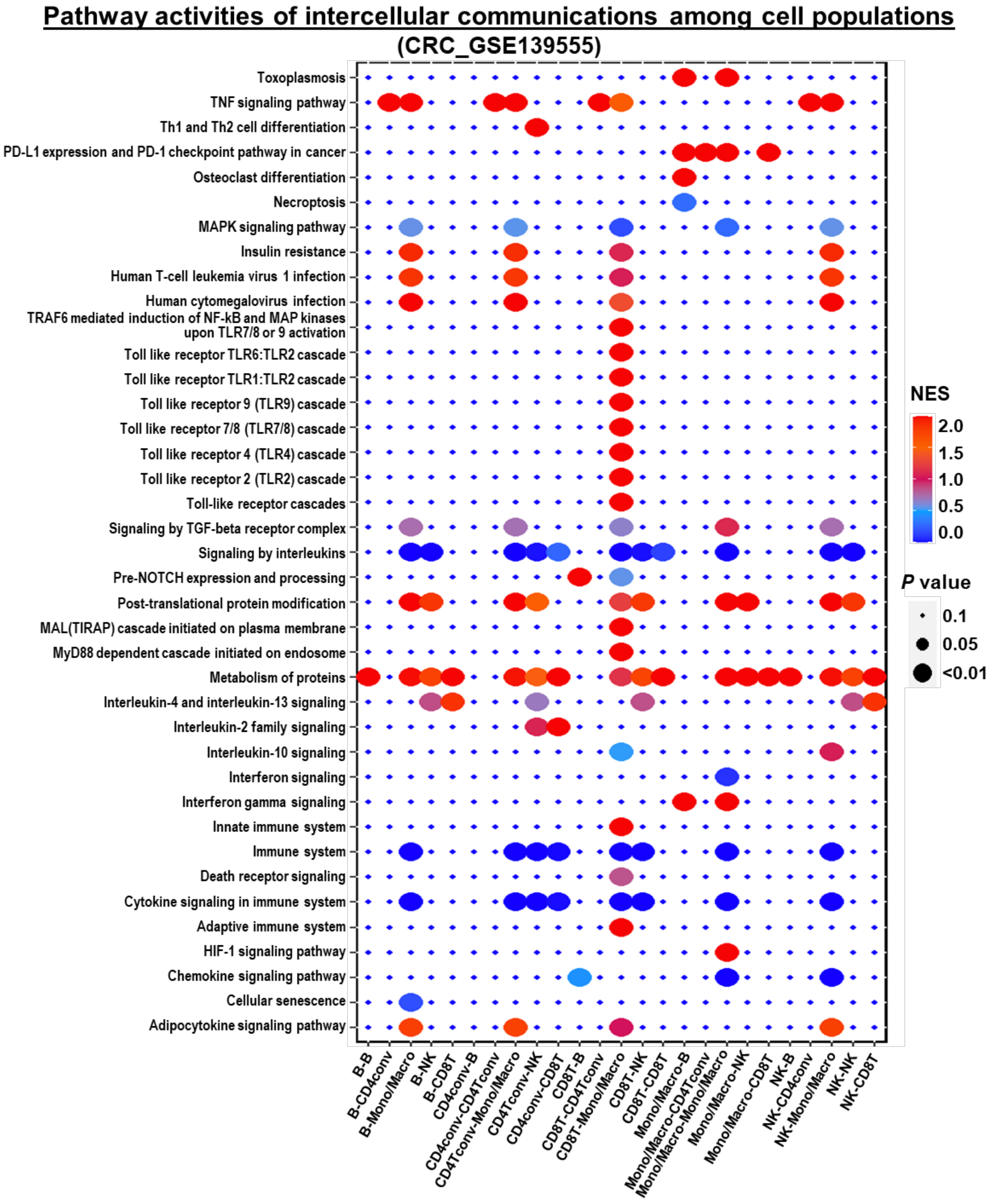

Fig. S6. Pathway activities of intercellular communications among cell populations of CRC_GSE139555: conventional $CD4^+$ T cells (CD4Tconv), $CD8^+$ T cells (CD8T), monocytes/macrophages (Mono/Macro), B cells (B) and natural killer cells (NK). Only pathways with $P < 0.000001$ in at least one cell populations were kept for visualization. NES, normalized enrichment score.

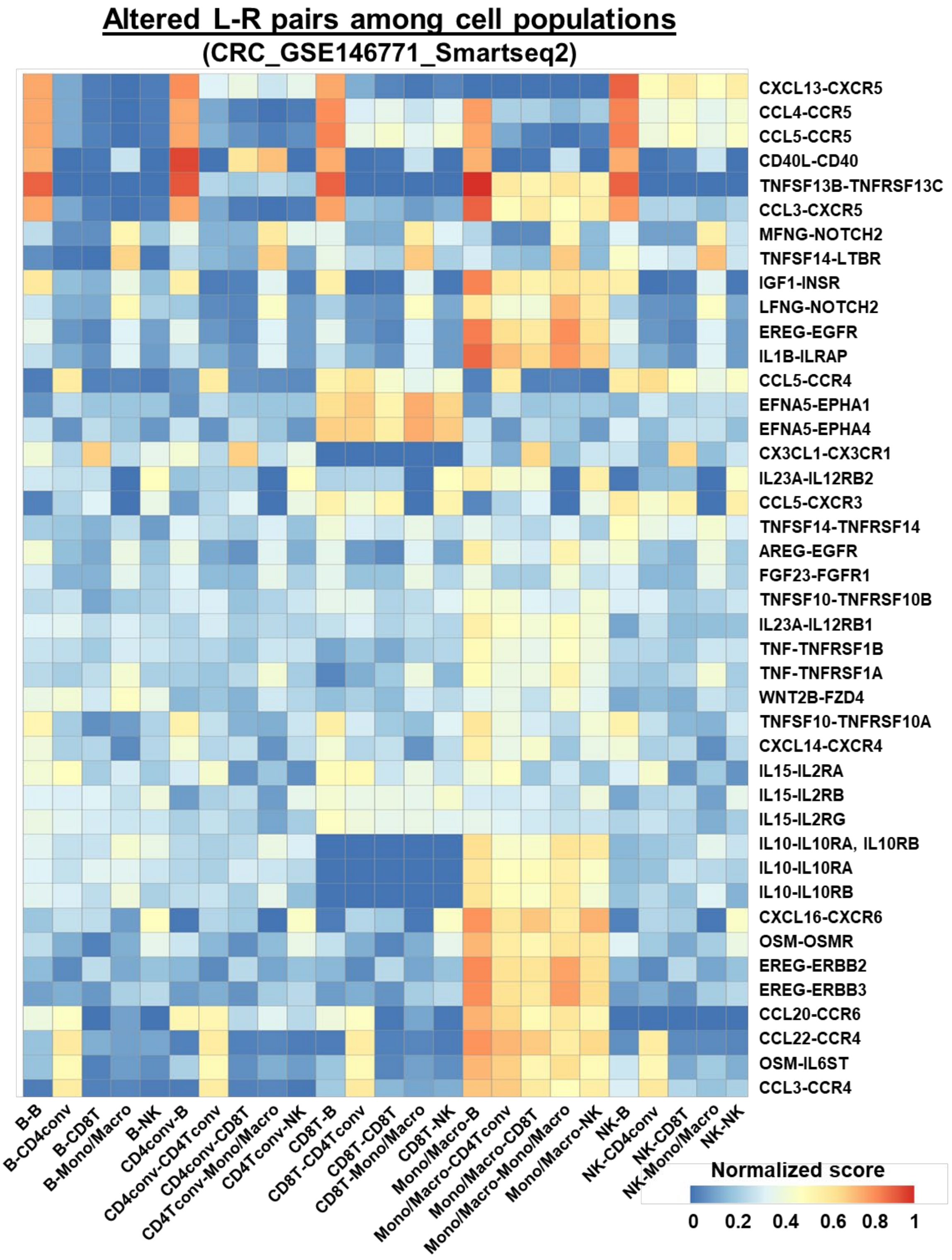

Fig. S7. Altered ligand (L)-receptor (R) pairs among cell populations for tumor-specific intercellular communications in CRC_GSE146771_Smartseq2: conventional CD4^{+} T cells (CD4Tconv), CD8^{+} T cells (CD8T), monocytes/macrophages (Mono/Macro), B cells (B) and natural killer cells (NK). Only transcription factors with GSEA *P*-adjusted values < 0.05 were kept for analysis. A cutoff to define correlated target genes for regulon was denoted as a correlation coefficient = 0.05.

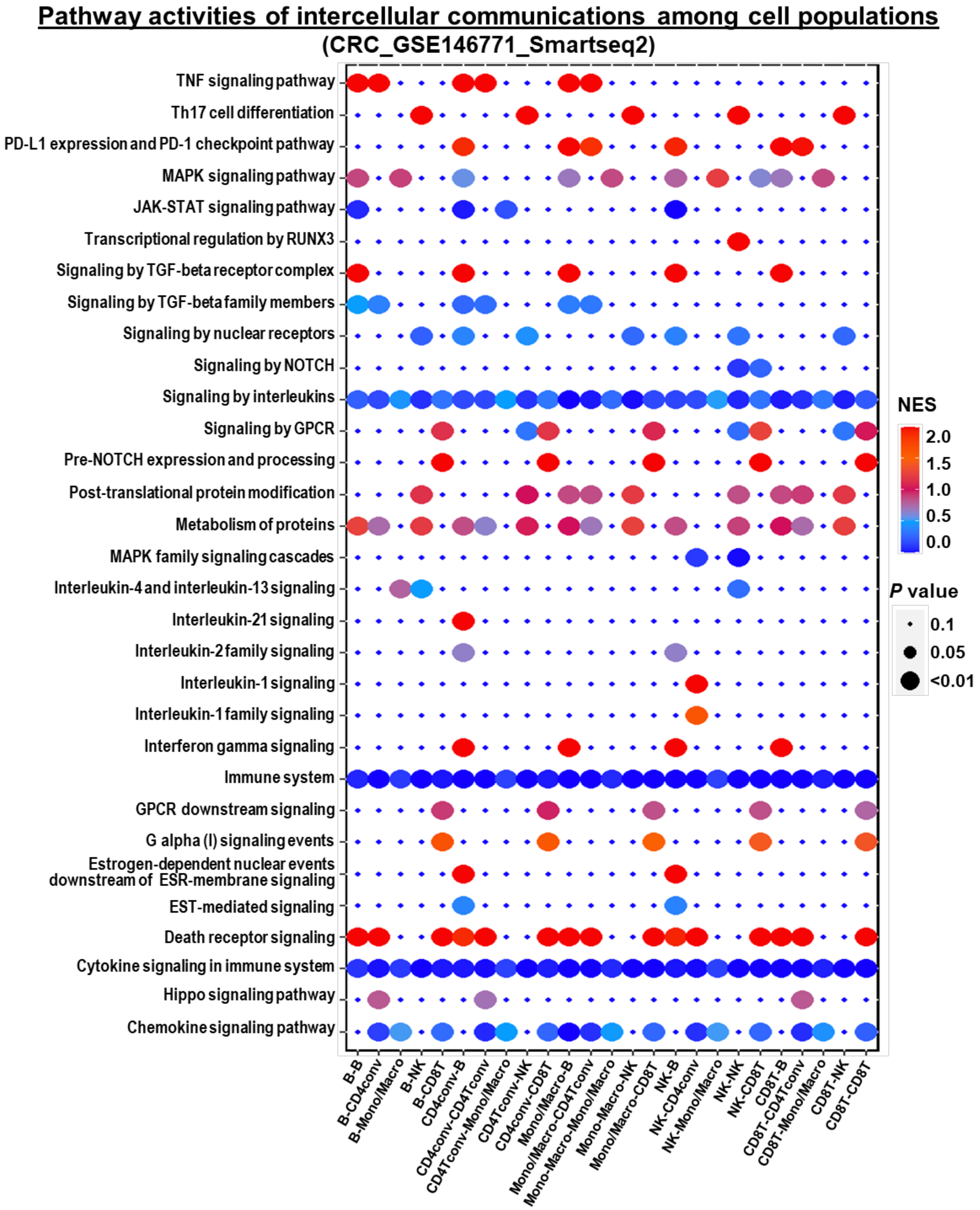

Fig. S8. Pathway activities of intercellular communications among cell populations of CRC_GSE146771_Smartseq2: conventional CD4+ T cells (CD4Tconv), CD8+ T cells (CD8T), monocytes/macrophages (Mono/Macro), B cells (B) and natural killer cells (NK). Only pathways with *P* < 0.000001 in at least one cell populations were kept for visualization. NES, normalized enrichment score.

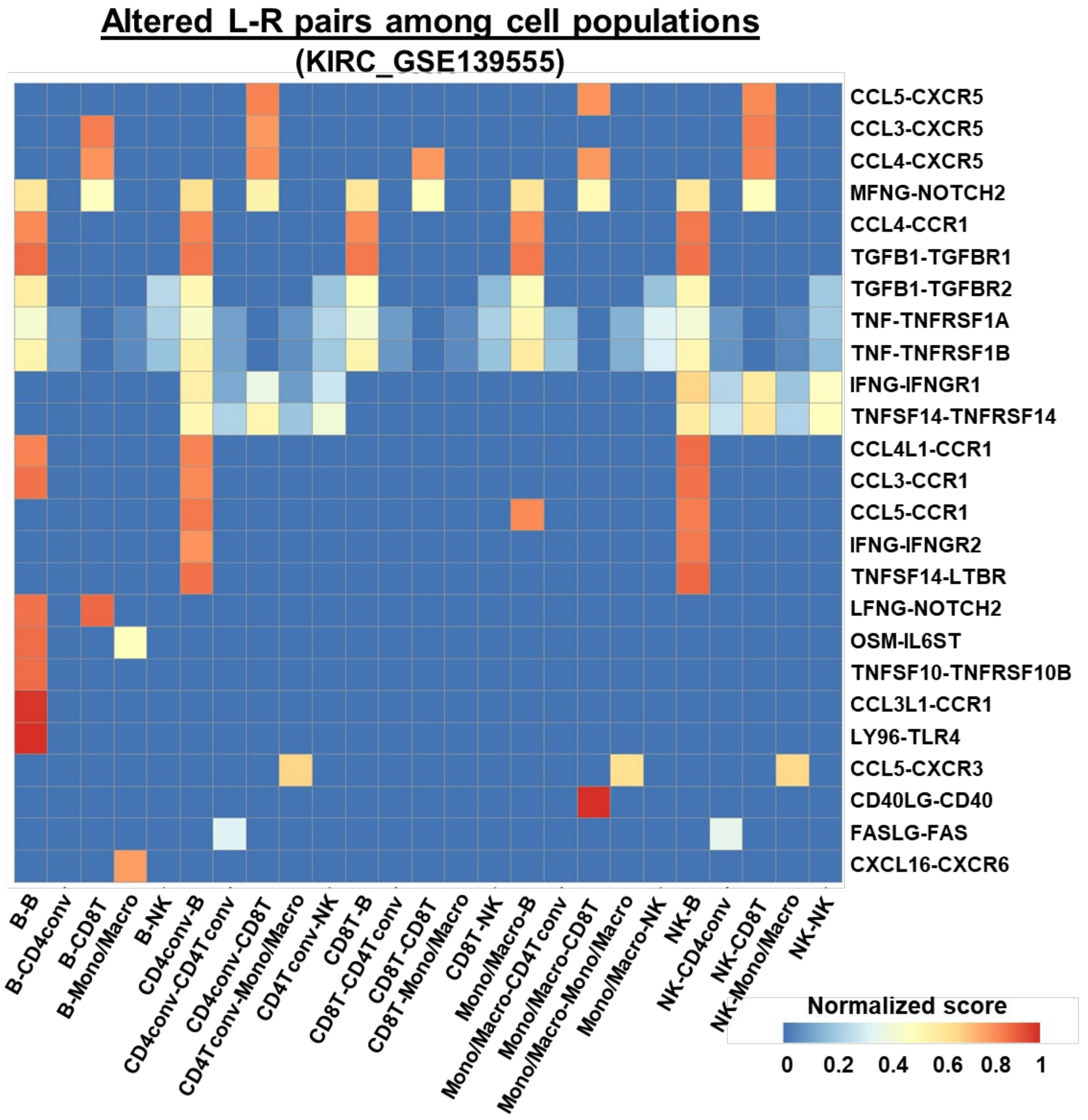

Fig. S9. Altered ligand (L)-receptor (R) pairs among cell populations for tumor-specific intercellular communications in KIRC_GSE139555: conventional $CD4^+$ T cells (CD4Tconv), $CD8^+$ T cells (CD8T), monocytes/macrophages (Mono/Macro), B cells (B) and natural killer cells (NK). Only transcription factors with GSEA *P*-adjusted values < 0.3 were kept for analysis. A cutoff to define correlated target genes for regulon was denoted as a correlation coefficient = 0.02.

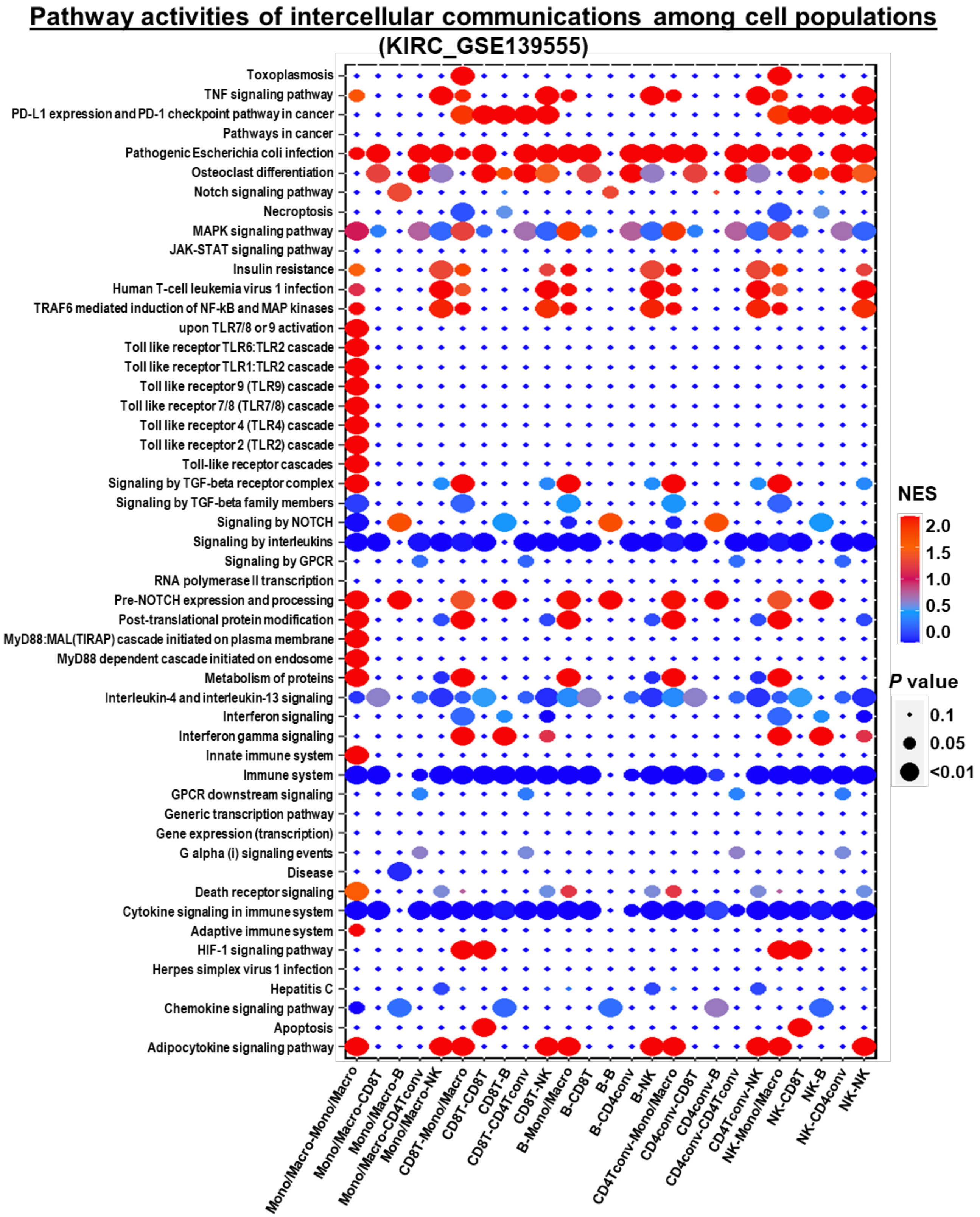

Fig. S10. Pathway activities of intercellular communications among cell populations of KIRC_GSE139555: conventional $CD4^+$ T cells (CD4Tconv), $CD8^+$ T cells (CD8T), monocytes/macrophages (Mono/Macro), B cells (B) and natural killer cells (NK). Only pathways with $P < 0.000001$ in at least one cell populations were retained for visualization. NES, normalized enrichment score.

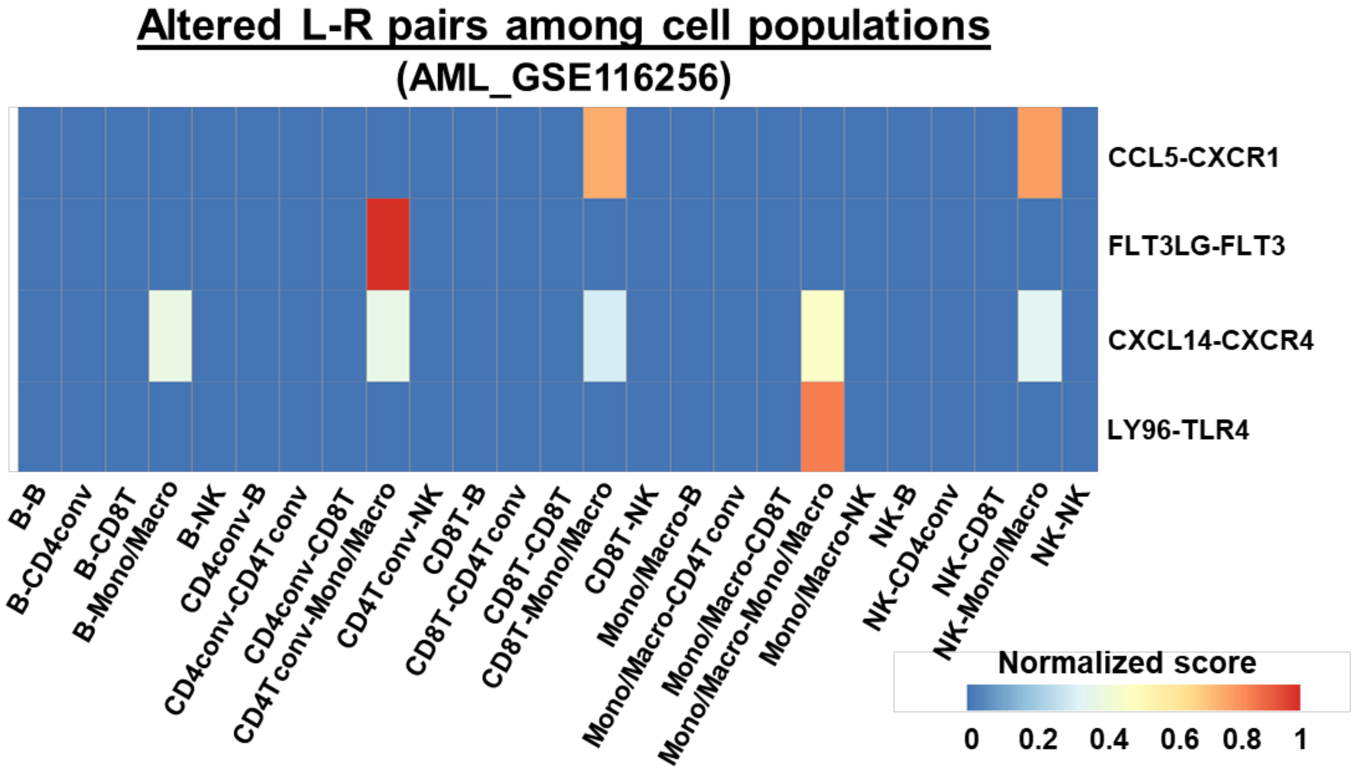

Fig. S11. Altered ligand (L)-receptor (R) pairs among cell populations in tumor-specific intercellular communications in AML_GSE116256: conventional $CD4^+$ T cells (CD4Tconv), $CD8^+$ T cells (CD8T), monocytes/macrophages (Mono/Macro), B cells (B) and natural killer cells (NK). Only transcription factors with GSEA *P*-adjusted values < 0.1 were retained for analysis. A cutoff to define correlated target genes for regulon was denoted as a correlation coefficient = 0.1.

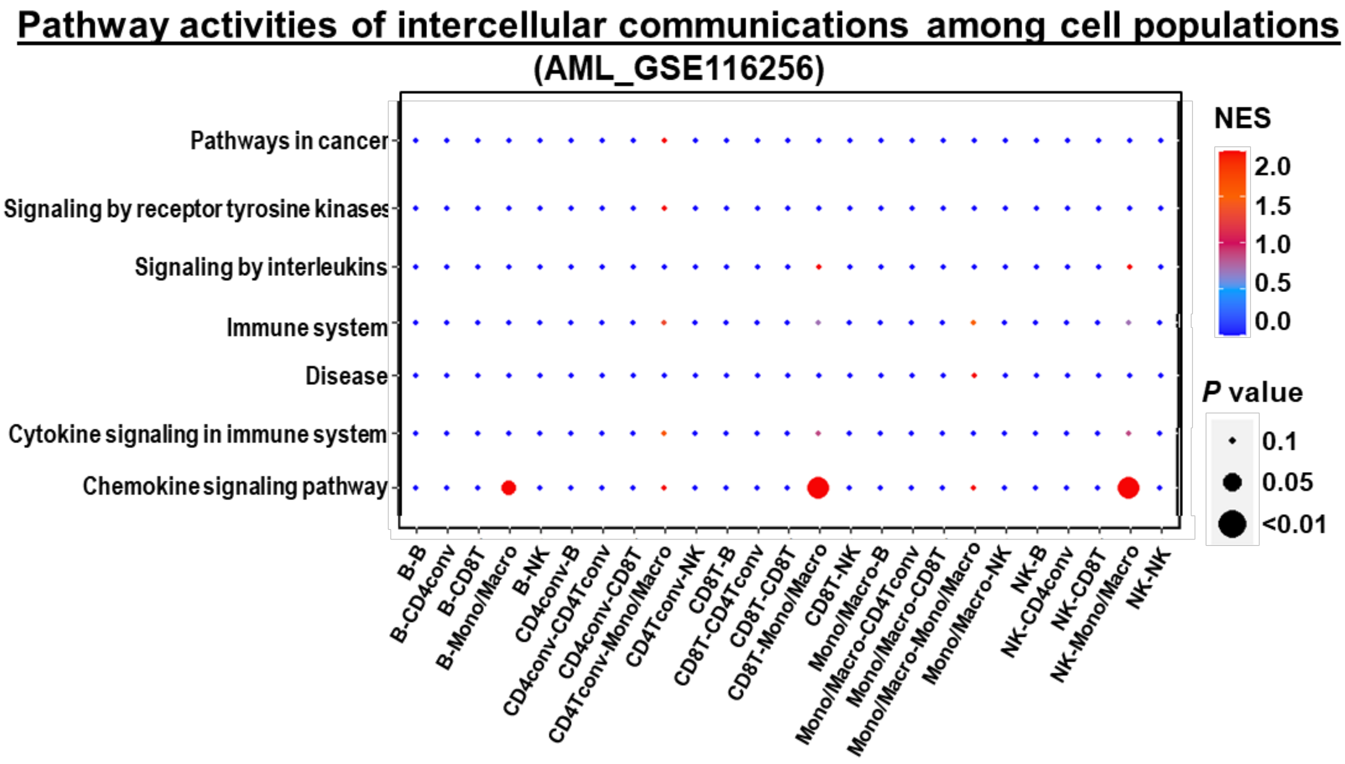

Fig. S12. Pathway activities of intercellular communications among cell populations of AML_GSE116256: conventional CD4$^+$ T cells (CD4Tconv), CD8$^+$ T cells (CD8T), monocytes/macrophages (Mono/Macro), B cells (B) and natural killer cells (NK) cells. Only pathways with $P < 0.1$ in at least one cell populations were retained. NES, normalized enrichment score.

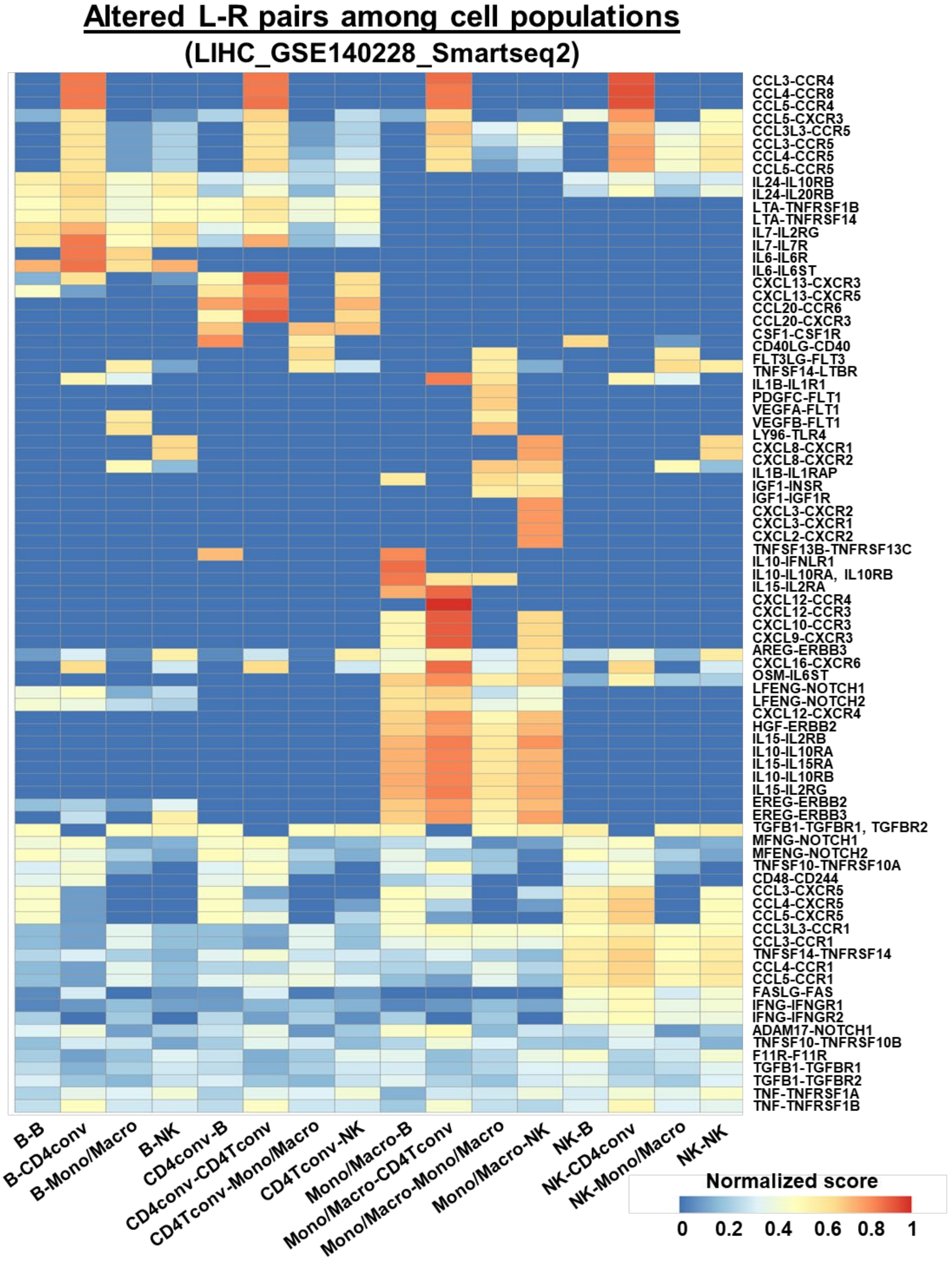

Fig. S13. Altered ligand (L)-receptor (R) pairs among cell populations in tumor-specific intercellular communications in LIHC_GSE140228_Smartseq2: conventional $CD4^{+}$ T cells (CD4Tconv), monocytes/macrophages (Mono/Macro), B cells (B) and natural killer cells (NK). Only transcription factors with GSEA *P*-adjusted values < 0.05 were kept for analysis. A cutoff to define correlated target genes for regulon was denoted as a correlation coefficient = 0.02.

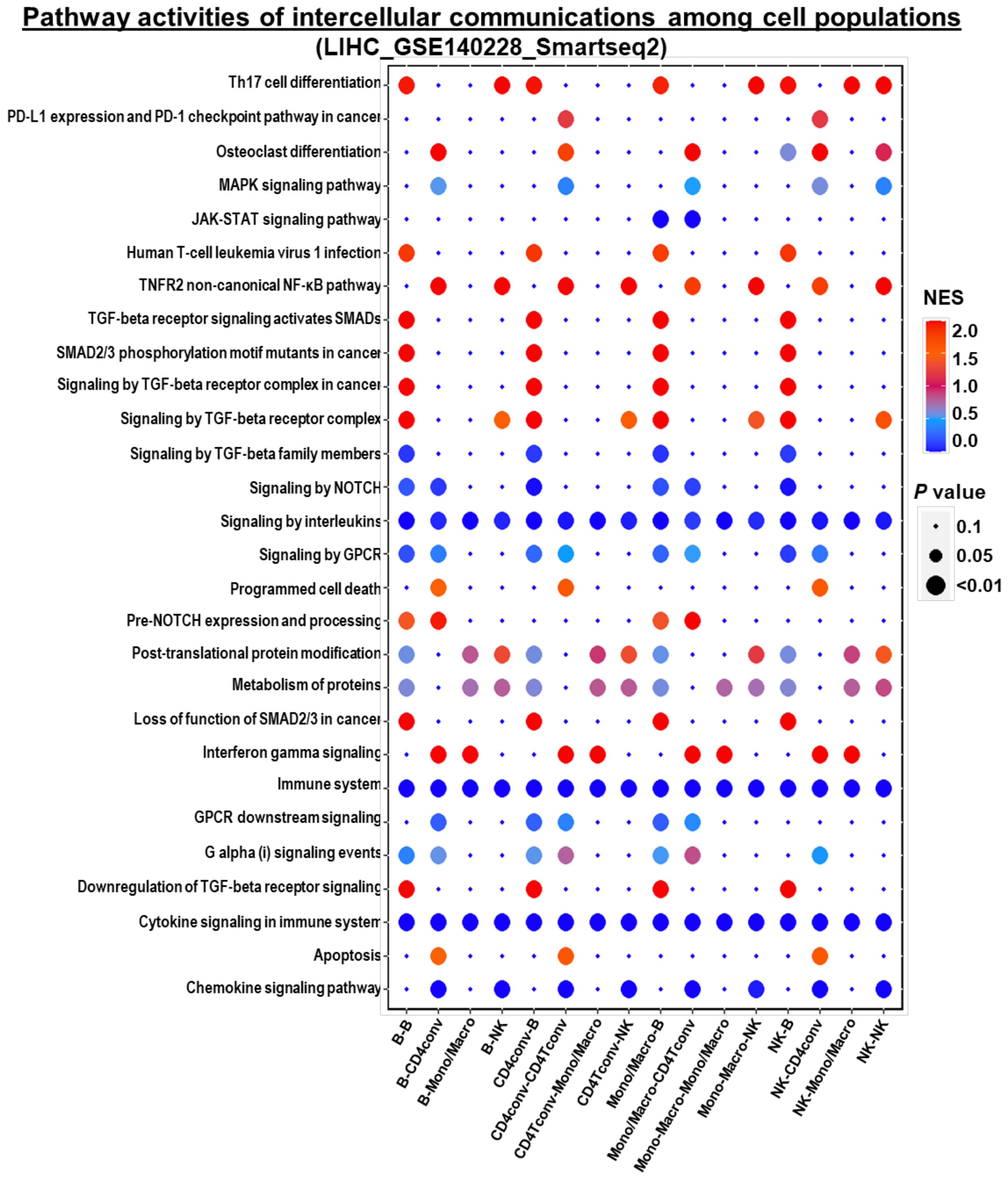

Fig. S14. Pathway activities of intercellular communications among cell populations of LIHC_GSE140228_Smartseq2: conventional $CD4^+$ T cells (CD4Tconv), monocytes/macrophages (Mono/Macro), B cells (B) and natural killer cells (NK). Only pathways with $P < 0.000001$ in at least one cell populations were retained for visualization. NES, normalized enrichment score.

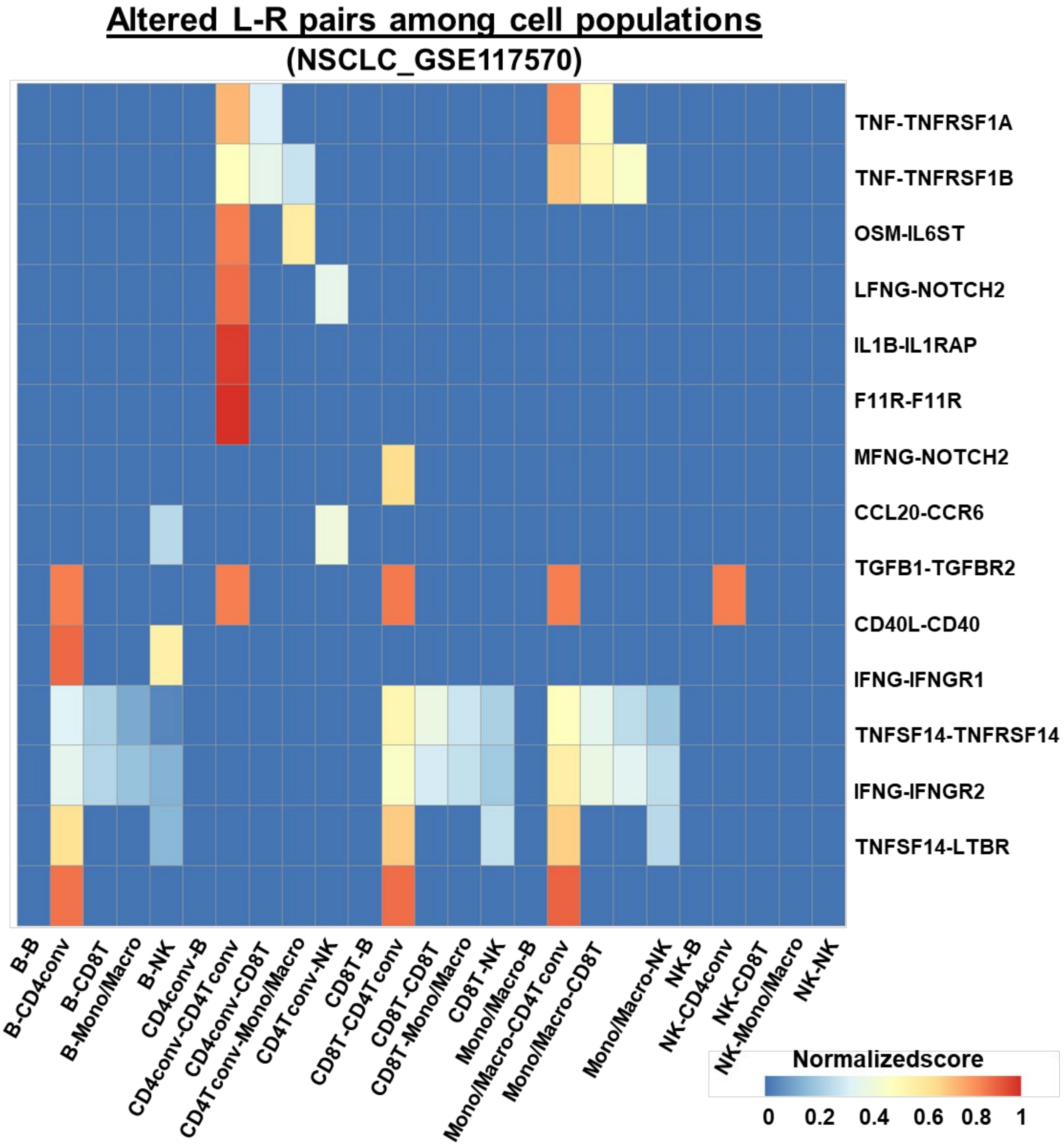

Fig. S15. Altered ligand (L)-receptor (R) pairs among cell populations in tumor-specific intercellular communications in NSCLC_GSE117570: conventional CD4$^+$ T cells (CD4Tconv), CD8$^+$ T cells (CD8T), monocytes/macrophages (Mono/Macro), B cells (B) and natural killer cells (NK). Only transcription factors with GSEA *P*-adjusted values < 0.05 were kept for analysis. A cutoff to define correlated target genes for regulon was denoted as a correlation coefficient = 0.1.

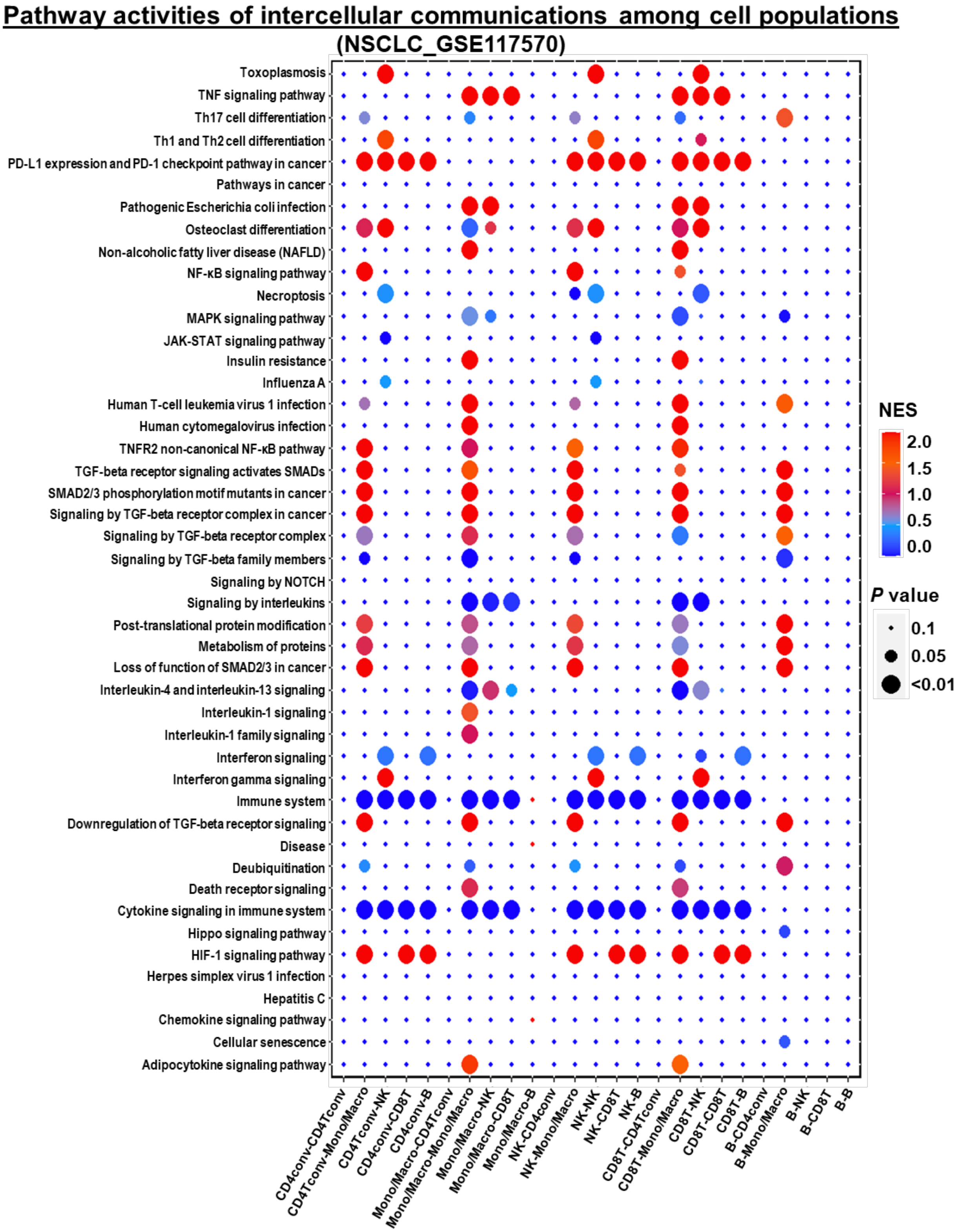

Fig. S16. Pathway activities of intercellular communications among cell populations of NSCLC_GSE117570: conventional $CD4^+$ T cells (CD4Tconv), $CD8^+$ T cells (CD8T), monocytes/macrophages (Mono/Macro), B cells (B) and natural killer cells (NK). Only pathways with $P < 0.1$ in at least one cell populations were retained. NES, normalized enrichment score.

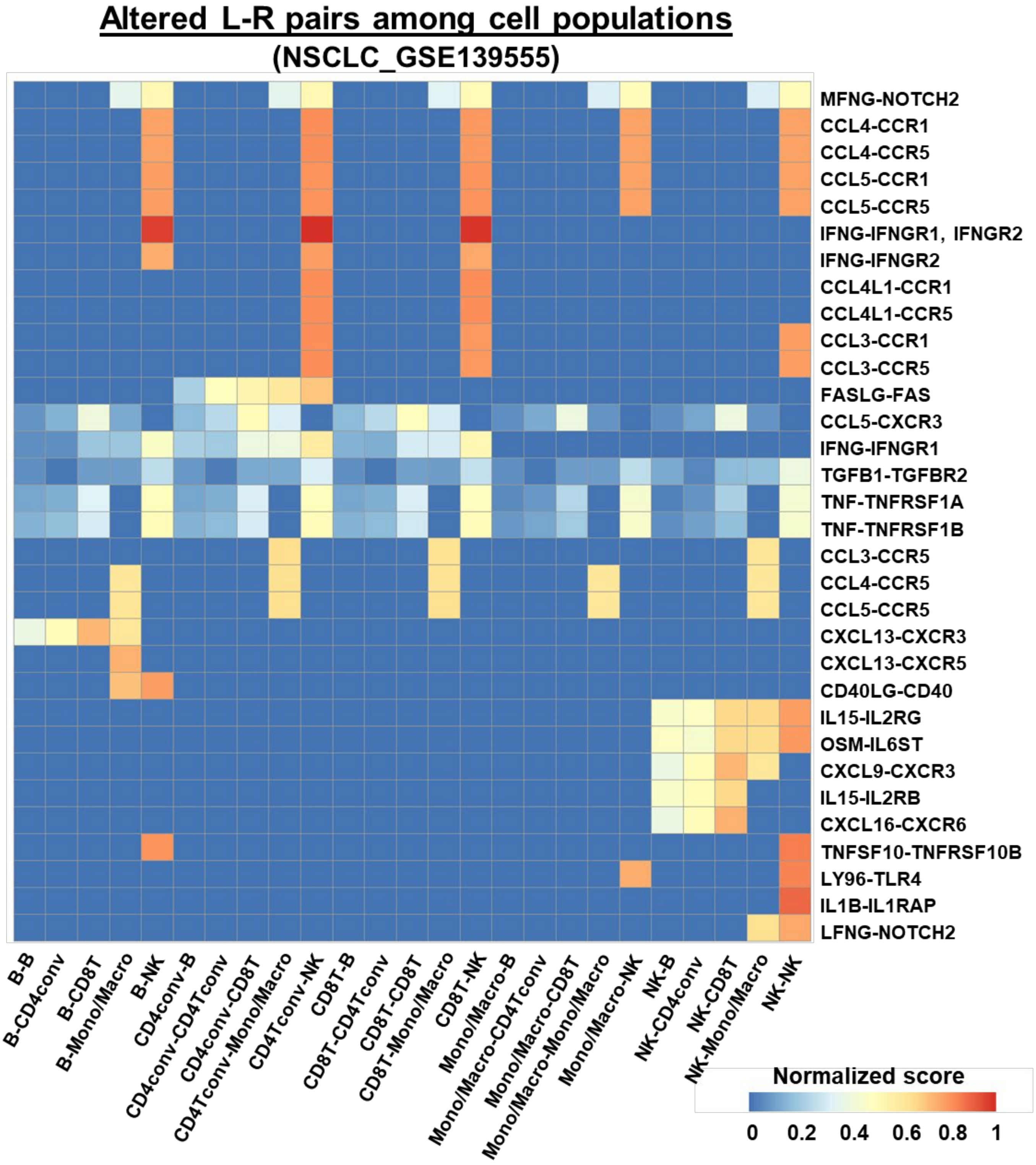

Fig. S17. Altered ligand (L)-receptor (R) pairs among cell populations in tumor-specific intercellular communications in NSCLC_GSE139555: conventional $CD4^+$ T cells (CD4Tconv), $CD8^+$ T cells (CD8T), monocytes/macrophages (Mono/Macro), B cells (B) and natural killer cells (NK). Only transcription factors with GSEA *P*-adjusted values < 0.3 were kept for analysis. A cutoff to define correlated target genes for regulon was denoted as a correlation coefficient = 0.02.

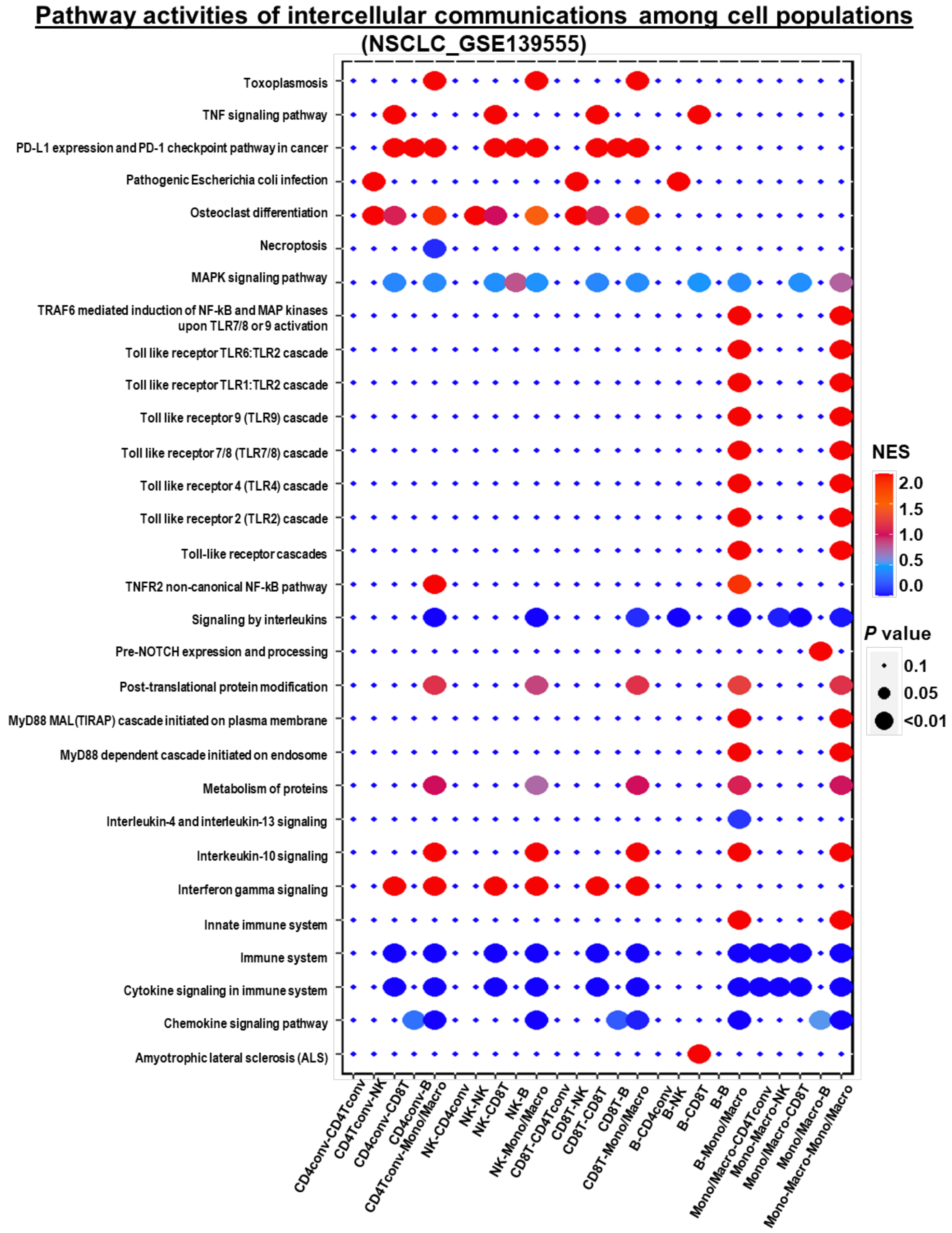

Fig. S18. Pathway activities of intercellular communications among cell populations of NSCLC_GSE139555: conventional CD4+ T cells (CD4Tconv), CD8+ T cells (CD8T), monocytes/macrophages (Mono/Macro), B cells (B) and natural killer cells (NK). Only pathways with *P* < 0.000001 in at least one cell populations were retained for visualization. NES, normalized enrichment score.

L-R pairs in the intercellular communications in DADA2 patients

NicheNet	CellCallEXT	NicheNet + CellCallEXT
CCL5-CCR7	EFNA4-EPHA4	CCL3L3-CCR1
HLA-E-CD8A	EFNB1-EPHB6	CCL3-CCR1
ITGA4-ITGB7	IFNG-IFNGR1	CCL4-CCR1
ITGB1-CD46	IFNG-IFNGR2	CCL5-CCR1
ITGB1-ITGA4	IL1B-IL1RAP	TGFB1-TGFBR2
ITGB2-ICAM2	IL15-IL2RB	
ITGB2-ITGAL	IL15-IL2RG	
PTPRC-CD2	IL15-IL15RA	
PTPRC-CD4	CXCL10-CXCR3	
SELL-SELPLG	LTA-TNFRSF1B	
SEMA4D-PTPRC	LTA-TNFRSF14	
ARF1-INSR	MFNG-NOTCH1	
CCL5-C5AR1	MFNG-NOTCH2	
HLA-E-PLXNB2	OSM-IL6ST	
HMGB1-LY96	F11R-F11R	
ITGB2-ICAM1	PF4-CXCR3	
LRPAP1-LRP1	CCL2-CCR1	
TGFB1-FCN1	CCL2-CCR2	
TGFB1-TLR2	CCL5-CXCR3	
ICAM1-IL2RG	ADAM17-NOTCH1	
ICAM1-SPN	TGFB1-TGFBR1	
ITGAM-ICAM2	TGFB1-TGFBR1,TGFBR2	
ADAM17-IL6R	TNF-TNFRSF1A	
APP-CD36	TNF-TNFRSF1B	
APP-LRP1	TNFSF14-LTBR	
APP-SORL1	TNFSF14-TNFRSF14	
APP-TGFBR2	TNFSF10-TNFRSF10B	
APP-TNFRSF14	TNFSF10-TNFRSF10A	
ICAM1-ITGAX	CD40LG-CD40	
ITGAM-ICAM1	CD48-CD244	
ITGAM-PLAUR	LY96-TLR4	
	LFNG-NOTCH1	
	LFNG-NOTCH2	

Fig. S19. Ligand (L)-receptor (R) pairs in intercellular communications in DADA2 patients identified by NicheNet and CellCallEXT.

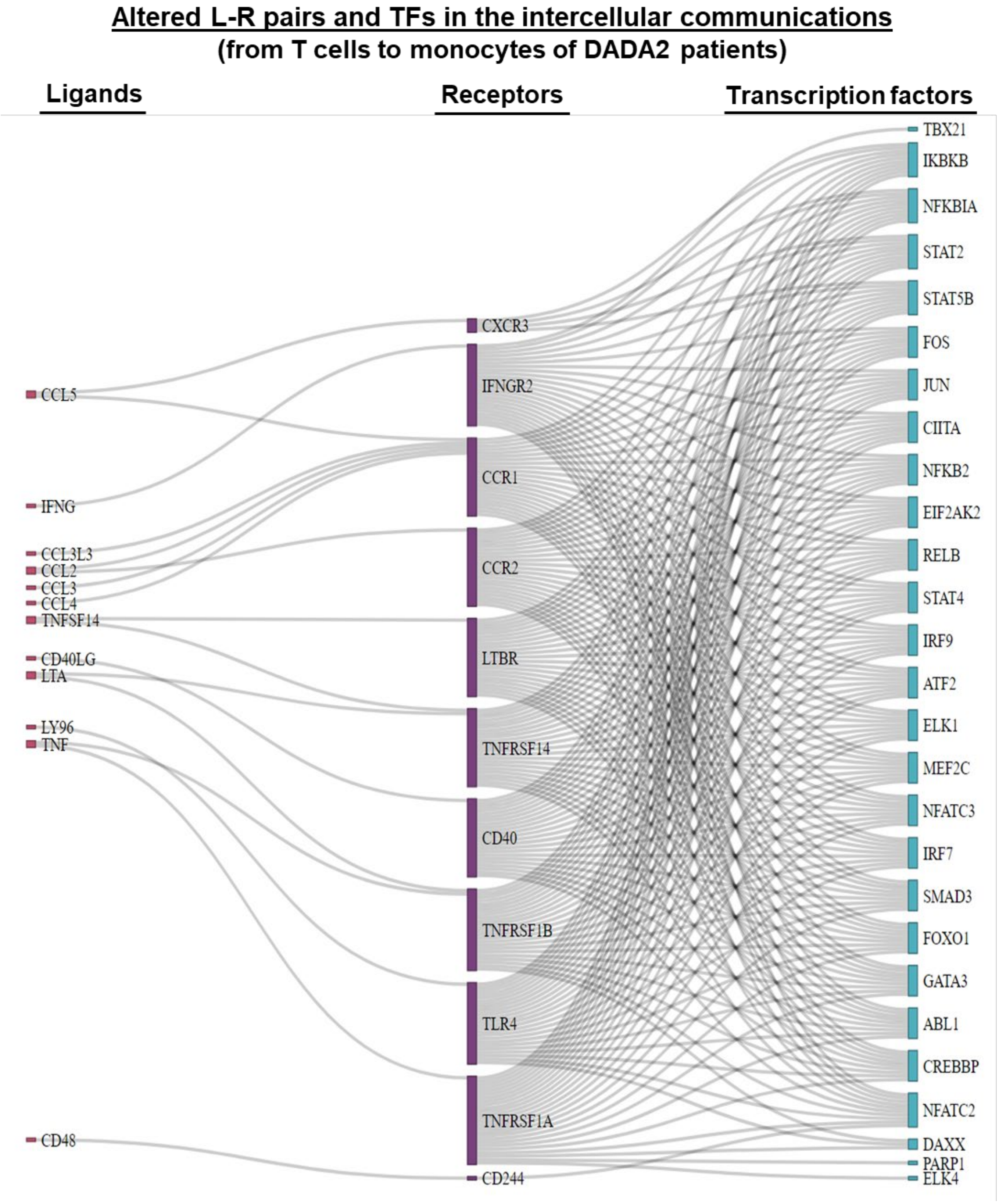

Fig. S20. Sankey plot of altered ligand (L)-receptor (R) pairs and downstream transcription factors (TFs) in the intercellular communications from T cells to monocytes of DADA2 patents.

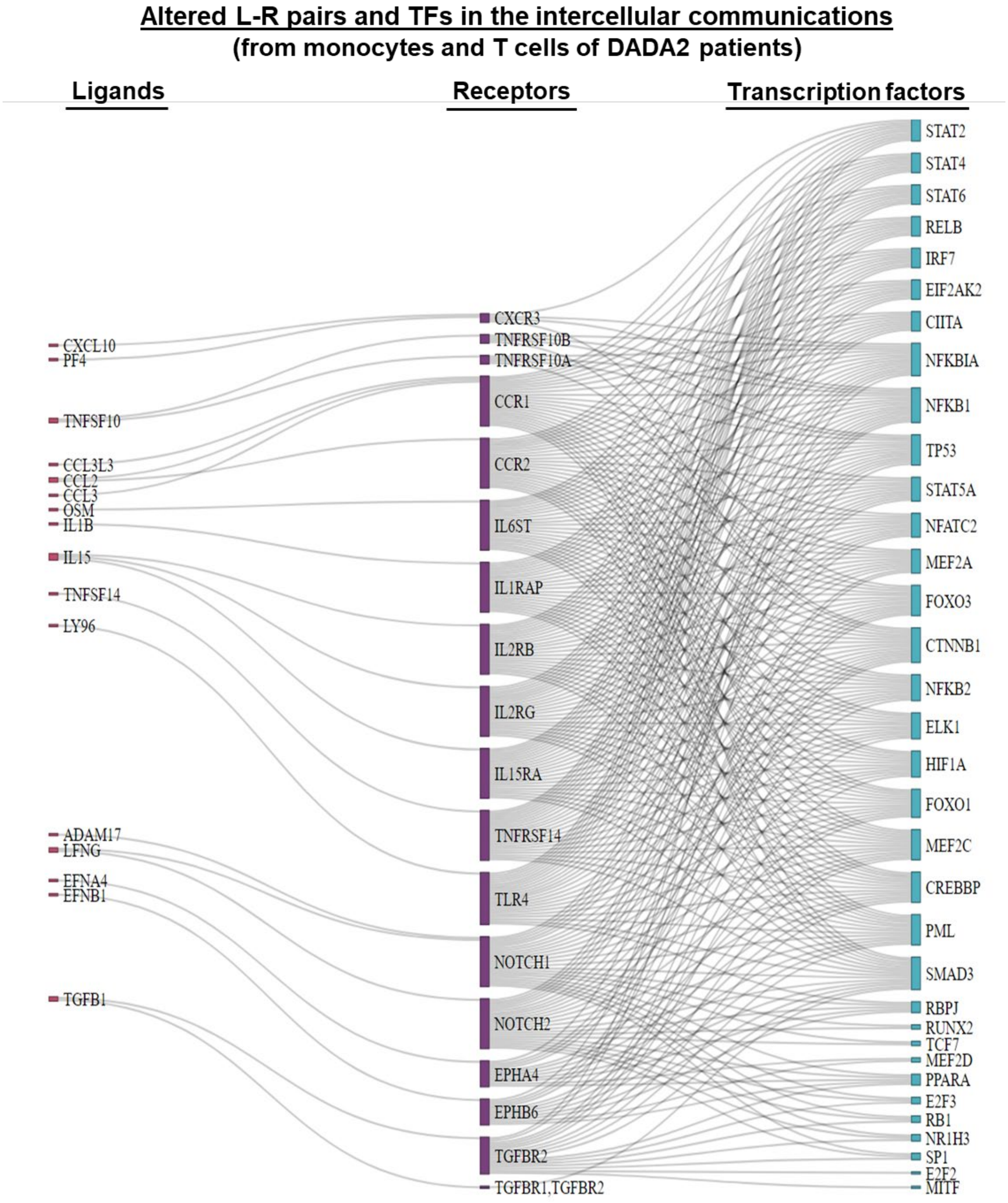

Fig. S21. Sankey plot of altered ligand (L)-receptor (R) pairs and downstream transcription factors (TFs) in intercellular communications from monocytes to T cells of DADA2 patents.
